# Integrating dark diversity, functional traits, and diagnostic species: a framework to diagnose bottlenecks in forest recovery

**DOI:** 10.3389/fpls.2026.1723617

**Published:** 2026-03-10

**Authors:** Ming-Hui Wang, Jian-Rong Su, Wan-De Liu, Shuai-Feng Li, Xiao-Bo Huang, Jia-Yan Shen, Rui-Guang Shang

**Affiliations:** 1 Institute of Highland Forest Science, Chinese Academy of Forestry, Kunming, China; 2 Pu’er Forest Ecosystem Research Station, National Forestry and Grassland Administration of China, Pu’er, China; 3 Pu’er Forest Ecosystem Observation and Research Station of Yunnan Province, Pu’er, China

**Keywords:** community completeness, dark diversity, early warning species, forest natural recovery, functional traits

## Abstract

**Introduction:**

Accurately assessing the natural recovery processes of forest ecosystems remains a key challenge in restoration ecology. The concept of dark diversity—the set of species absent from a site but belonging to its habitat-specific species pool—provides a novel lens for this assessment.

**Methods:**

In this study, we developed and applied an integrated diagnostic framework that synthesizes dark diversity, functional traits, and diagnostic species. We applied this framework to a chronosequence of recovering forest ecosystems in subtropical China, representing early, middle, and late recovery stages.

**Results:**

Our results demonstrated that the Community Completeness Index (CCI), derived from dark diversity, increased significantly during recovery, with its stabilization indicating the approach to a stable state. The framework identified stagespecific early-warning species: the absence of light-demanding, acquisitive transitional species in the mid-stage signaled successful progression, while the absence of shade-tolerant, conservative climax species in the late-stage signaled potential degradation. Crucially, analysis using Dark Diversity Affinity (DDA) revealed that the functional traits of species (e.g., seed mass, mycorrhizal type, leaf economics) were the primary filters determining species absence, exhibiting a stronger influence than local environmental conditions. These filters shifted predictably across stages, from dispersal and establishment limitations early on to competitive interactions later.

**Discussion:**

The proposed framework translates dark diversity theory into an actionable tool for restoration. It moves beyond simple observation to diagnose recovery success, pinpoint specific bottlenecks, and inform targeted interventions such as assisted dispersal or canopy management. This provides a mechanism-based approach for guiding precision restoration in forest ecosystems.

## 1 Introduction

Forests represent one of the most important ecosystems on earth and play an irreplaceable role in biodiversity protection, climate regulation, and maintenance of carbon and water cycles (Brockerhoff et al., 2017; Chen et al., 2022). In recent decades, population growth and social development have disturbed and damaged the environment, leading to the degradation of forest ecosystems (Hosseini et al., 2024; Liu et al., 2024; Riedl et al., 2024). Forest degradation weakens the ecosystem’s service functions, thus affecting people’s living environment (Liu et al., 2024). Therefore, it is important to reconstruct and restore degraded forest ecosystems. In terms of current conditions, natural recovery and artificial restoration are the two most suitable methods (Qin et al., 2022; Zhang et al., 2024a). Among them, natural recovery mainly relies on natural forces to restore degraded forest ecosystems. Compared to costly and technically complex artificial restoration, it is usually more cost-effective and easier to apply on a large scale (Zhang et al., 2024a). Although natural recovery has great potential, it still faces many problems in practical application, such as the uncertainty of recovery effects and processes, and the difficulty in establishing dominant species during natural recovery (Zhao et al., 2023; Zhang et al., 2024a). Given this, most strategies for restoring forest ecosystems are primarily based on natural recovery, with artificial restoration used to assist natural recovery (Atkinson and Bonser, 2020).

Assessing the natural recovery potential of forest ecosystems is the first step in ecological restoration work (Gatica-Saavedra et al., 2017). Such assessment determines whether recovery objectives are being met, identifies facilitating or limiting factors, and informs decisions on the necessity, timing, and methods of artificial restoration. Traditional assessments often rely on observed diversity metrics, requiring a suitable reference ecosystem (usually pre-degradation) and potentially overlooking the full spectrum of potential biodiversity (Helm et al., 2015; Shackelford et al., 2013; Chollet et al., 2025). The concept of “dark diversity” - the set of species that are absent from a site but belong to its habitat-specific species pool under suitable conditions (Pärtel et al., 2011) offers a novel and more comprehensive perspective. Integrating dark diversity into restoration ecology can enhance the assessment of natural recovery potential, identify factors limiting species occurrence, and refine management priorities (Moeslund et al., 2017; Deschênes et al., 2024; Feng et al., 2025).

Hitherto, some studies have successfully applied the concept of dark diversity to restoration and conservation issues. First, the Community Completeness Index (CCI) based on dark diversity can be used to assess whether a natural ecosystem in recovery is approaching successful restoration (Cam et al., 2000; De Bello et al., 2012; Pärtel et al., 2013). Lower CCI indicate that the current ecosystem lacks a large number of suitable species and still requires further restoration, while higher CCI suggest that the ecosystem’s recovery has been relatively successful (Pärtel et al., 2013; Moeslund et al., 2017; Deschênes et al., 2024). Secondly, combine the set of species with high dark diversity and diagnostic species (species that indicate specific habitat types, ecological conditions, or specific characteristics of biological communities) to obtain a set of early warning species for the current ecosystem. By analyzing the characteristics of these early warning species, it can be inferred what potential problems may exist in the natural recovery process of ecosystems and provide reasonable restoration guidance (Caro and Girling, 2010; Nicod et al., 2019; Rodríguez-Rojo et al., 2020; Zanzottera et al., 2020). Finally, establishing the link between dark diversity and plant functional traits and environmental characteristics, identifying limiting factors in the natural recovery process of ecosystems, and assisting in the reconstruction of species in dark diversity (Violle et al., 2007; Hemrová and Münzbergová, 2015; Boussarie et al., 2018; Carlucci et al., 2020; Hostens et al., 2023; Aubin et al., 2024).

Despite these conceptual advancements, a critical knowledge gap remains: the lack of a unified, operational framework that synthesizes these diagnostic tools into actionable guidance for forest restoration. To address this gap, we integrate dark diversity theory into a comprehensive framework for assessing forest natural recovery (**Supplementary Figure 1**, which outlines the conceptual structure and operational stages of our integrated diagnostic tool). We applied this framework to forest ecosystems at different recovery stages in southern subtropical region of Yunnan Province, China. *Pinus kesiya* forests, as prevalent secondary successional communities with dynamic understory turnover, serve as an ideal system for investigating species re-establishment and the role of dark diversity in community development. Our specific research objectives are: (1) to assess the natural recovery of forest ecosystems using CCI without a reference community for recovery evaluation; (2) to identify key issues existing during the recovery process of forest ecosystems through early warning species; (3) to clarify the formation mechanisms of dark diversity and identify key factors hindering forest ecosystems at different recovery stages. By synthesizing these objectives, this study aims to translate complex ecological diagnostics into precise, stage-specific restoration strategies, thereby advancing the development of resilient and high-biodiversity forest ecosystems.

## 2 Materials and methods

### 2.1 Study region

**Figure 1:**
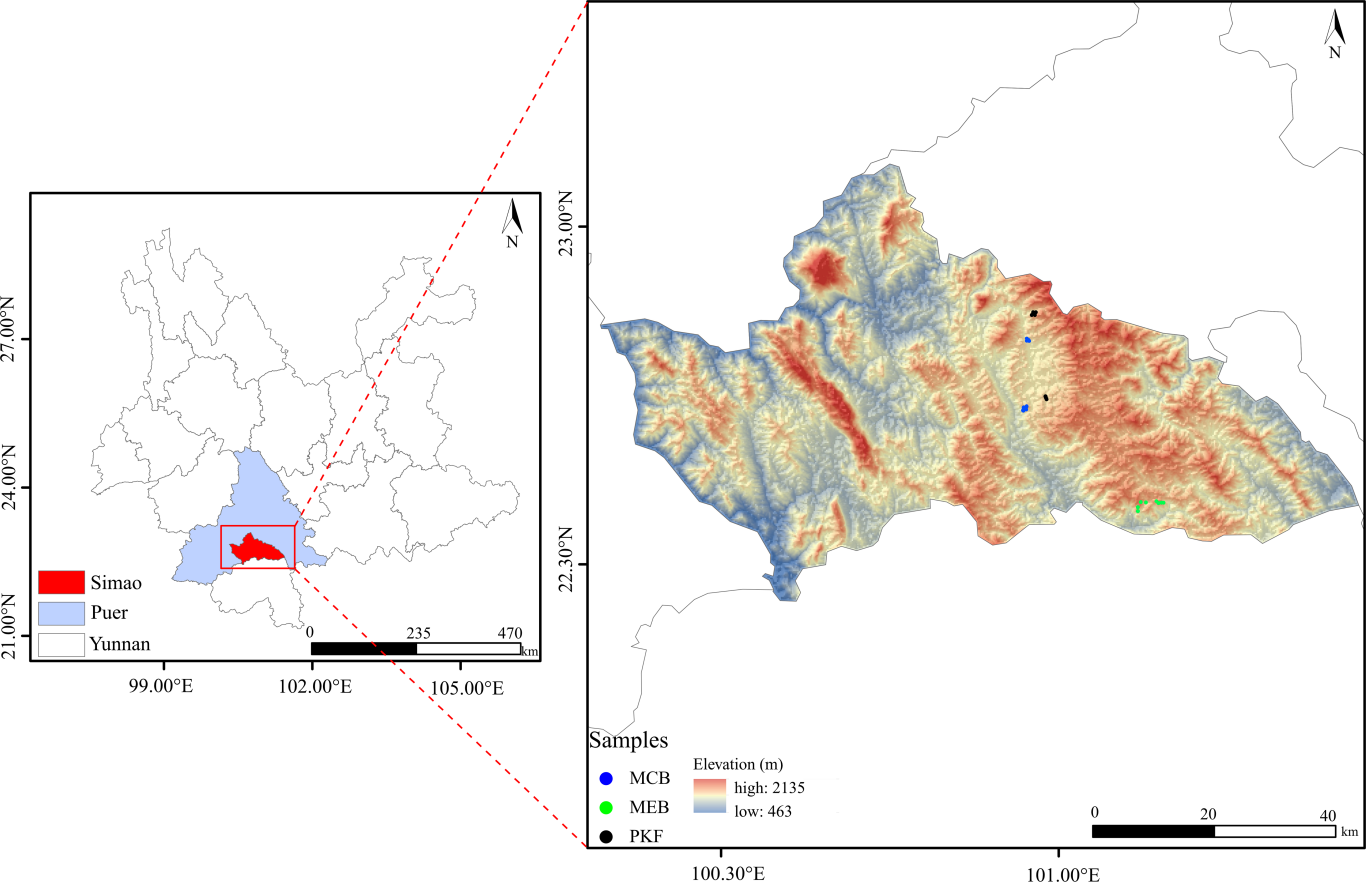
Distribution diagram of the study region and sampling points.

This study was conducted in Simao District, Pu ‘er City, Yunnan Province (22°34′–22°53′N, 100°56′–100°09′E), China (**Figure 1**). This region is located within the boundary and transition zone between the tropical zone and the southern subtropical zone. The climate is tropical humid, with an average annual temperature of 21.7°C and an average annual precipitation of 1490 mm. Influenced by the southwestern Indian Ocean monsoon, rainfall is concentrated in summer and autumn, while winter and spring are relatively dry. The main soil type in the study region is acidic laterite soil (Shang et al., 2021). The main vegetation types in the study area are the *Pinus kesiya* forest, mixed coniferous and broadleaf forest, and monsoon evergreen broad-leaved forest. Most *P. kesiya* forests are secondary forests formed after human disturbance (e.g., logging and farming) of original monsoon evergreen broad-leaved forests. Through natural regeneration, these gradually develop into mixed coniferous and broadleaf forests. This well-documented successional trajectory for southwest Yunnan forms the empirical basis for our classification of forest recovery stages (Tang, 2010). The dominant tree species include *Castanopsis hystrix*, *Castanopsis echidnocarpa*, and *Schima wallichii*. The shrub layer consists mainly of tree saplings, and the herb layer is dominated by *Scleria levis*, *Dicranopteris pedata*, and some ferns (Tang, 2010).

### 2.2 Sample site setting and investigation

To investigate forest natural recovery across different stages, plots were established within three distinct forest types in Simao District, Pu’er City: Natural Secondary *Pinus kesiya* var. *langbianensis* Forest (PKF), Natural Mixed coniferous and broadleaf forest (MCB), and Natural Monsoon Evergreen Broadleaf Forest (MEB). These three forest types were selected to represent empirically recognized early, middle, and late stages of natural recovery, respectively (Tang, 2010). By evaluating tree rings and consulting historical documents, the ages of the three types of forest communities are approximately 40, 70, and 90 years old. The determination of the three forest types is based on an integrated assessment of canopy species and species with relative importance greater than 10% in the community. To ensure comparability and minimize confounding environmental factors, all 24 plots were situated within a contiguous geographical area characterized by consistent climatic conditions and the same acidic laterite soil type. Plots were specifically chosen to exhibit similar general topographical conditions and comparable historical land-use backgrounds within each successional category, specifically restricted to an altitude range of 1317–1648 m, slopes between 6° and 18°. Detailed topographic data for survey plots at different stages of forest restoration, including specific ranges of altitude and slope, are provided in **Supplementary Table 1**. Following the Center for Tropical Forest Science (CTFS) standards (Condit, 1995), eight 30 m×30 m plots were established for each forest type, with a minimum distance of 100 m between plots, totaling 24 plots. The sample size of eight replicates per stage was chosen to ensure adequate representation of intra-stage structural variability while maintaining logistical feasibility for high-intensity woody plant censuses, consistent with previous successional studies in this region (Shang et al., 2021). Geographic coordinates, altitude, slope, and aspect were recorded for each plot. Within each plot, all woody plants with a diameter at breast height (DBH) ≥ 1 cm were identified and counted, including lianas, shrubs, and trees. Based on the survey results, we calculated the forest density (FD), the ratio of tree number to plot area. We also used the allometric growth Equation 1 to calculate the total aboveground biomass (AGB) of woody plants with DBH ≥1 cm in each 30 m×30 m plot (Zhang et al., 2024b).

(1) AGB=Exp[−2.334+2.118ln(DBH)+0.5436ln(H)+0.5953ln(WD)]

where AGB represents total aboveground biomass (kg), DBH is the diameter at breast height (cm), H is the actual measured height (m), and WD is stem wood density of species (g·cm^−3^).

During the community survey, soil samples (0–20 cm depth, below the litter layer) were collected using a stainless-steel soil corer. Five cores per plot were composited, sieved (2 mm mesh) to remove roots and stones, and analyzed indicators included soil pH, soil water content (WCOS), soil nutrients and enzyme activities. Soil nutrients included soil organic carbon (SOC), total nitrogen (TN), total phosphorus (TP), total potassium (TK), alkali-hydrolysable nitrogen (HN), available phosphorus (AP), and available potassium (AK). Enzyme activities assessed were urease (Ure), β-glucosidase (BG), β-1,4-N-acetylglucosaminidase (NAG), cellulase (CBH), and acid phosphatase (ACP). Analytical methods followed Shang et al. (2021, 2023) and Deforest (2009). The unit of Ure measurement is mg·g^-^¹ 24h^-^¹, while the units for other enzymes are nmol·g^-^¹ h^-^¹.

Nine plant functional traits related to the plant’s morphology, reproduction, dispersal, population attributes, resource strategy, and life history were compiled (Li and Prentice, 2024): mycorrhizal associations, leaf carbon content (LOC), leaf nitrogen content (LTN), leaf phosphorus content (LTP), leaf area (LA), specific leaf area (SLA), leaf dry matter content (LDMC), stem and wood density (WD), and seed mass (SM). Mycorrhizal types were determined based on the published literature and online datasets (http://mycorrhizas.info/index.html) (Soudzilovskaia et al., 2020). To ensure that trait values accurately reflect local environmental conditions and minimize the impact of regional-scale intraspecific variation, these quantitative functional traits (except seed mass) were directly measured within the study area (Pu’er region). For seed mass, data were primarily obtained from the Germplasm Bank of Wild Species (http://www.genobank.org); although these data came from different study plots, the sampling sites were also located within the Pu’er region. Since the number of sampling points for seed mass per species was typically small (ranging from 1 to 3), average values were used for calculations. Public databases, such as the TRY Plant Trait Database, LEDA Traitbase, and Seed Information Database (SID), were only utilized for a few rare species with limited local available records (Kleyer et al., 2008; Kattge et al., 2011; Liu et al., 2019).

### 2.3 Data analysis

#### 2.3.1 Dark diversity and community completeness

We constructed a metacommunity using the combined species from all 24 plots across three recovery stages. Which each observation site was treated as a local community and the combination of all sites formed the metacommunity. The metacommunity matrix consisted of observation sites (rows) and species (columns), where the species are observed at least once in the metacommunity. Dark diversity was estimated from species co-occurrence patterns within each metacommunity dataset using R package ‘DarkDiv’ (De Bello et al., 2016; Carmona and Pärtel, 2021; Hostens et al., 2023; Paganeli et al., 2024). Specifically, we used the hypergeometric method available in the R package for estimation. This method uses the hypergeometric distribution to calculate the expected number of co-occurrences of species pairs under randomness (given by the mean of the hypergeometric distribution), standardizes the difference between this expected value and the observed co-occurrence frequency, and then averages and probabilizes it, ultimately obtaining a probability value that is independent of regional frequencies and represents the specific ecological suitability of each species at a particular location (Carmona and Pärtel, 2021). The summed suitability probability values of all missing species at a certain location are referred to as the site dark diversity, and the average probability values of each species being missing during the same recovery phase are referred to as the species dark diversity (Pärtel et al., 2013; Tang et al., 2023; Chollet et al., 2025). The CCI is the ratio of species richness to total site dark diversity, expressed in logarithmic form ln (observed diversity/dark diversity) (Pärtel et al., 2013; Tang et al., 2023). To evaluate the comparison of CCI with traditional recovery success indicators, we used ANOVA and Tukey’s *post-hoc* test to examine differences in observed diversity, dark diversity, and community completeness across different recovery stages. We also assessed the relationship between CCI and species richness using linear regression.

#### 2.3.2 Identification of early warning species

First, we identified the diagnostic species for each stage. Instead of using traditional abundance-based IndVal, we used the Functional Association Index (ϕ) (Ricotta et al., 2020). We calculated the abundance-weighted Community Weighted Mean (CWM) for each stage to represent its functional center. Before distance calculation, all traits were log-transformed and standardized using Z-scores to ensure equal weighting. The functional similarity between each species and the functional center was calculated as 1−dnorm, where dnorm is the Euclidean distance in multidimensional trait space normalized by the maximum observed distance (Ricotta et al., 2020). The index ϕ was then calculated as the product of a species’ relative abundance in a stage and its functional similarity to that stage’s center. This approach ensures that diagnostic species are those that are both functionally compatible with and characteristic of a specific recovery stage. Significance was tested through 999 permutations (p< 0.05). Second, we defined the dark diversity set. Following Dalle Fratte et al. (2022), species with a dark diversity probability ≥ 0.6 were selected. This specific threshold was used to minimize the statistical uncertainty associated with a probability of 0.5, ensuring that the missing species have a clear ecological affinity for the site. Finally, the intersection of the diagnostic species set and the dark diversity set was defined as the “early-warning species set”. To ensure the robustness of our results, we verified the assumption that dark diversity is not biased toward diagnostic species through a permutation-based null model. Specifically, the null model was constructed by randomly shuffling species identities in the trait matrix 999 times while keeping the community’s functional center constant, thereby testing whether the observed association between species and specific recovery stages was significantly greater than expected by chance.

#### 2.3.3 Dark diversity affinity

Considering that species and sites can independently form dark diversity patterns, directly associating dark diversity with environmental characteristics or functional traits may confuse their interactions. Here, we introduce a new index proposed by Fujinuma and Pärtel (2023), called dark diversity affinity (DDA). This index measures the tendency of species and sites to increase dark diversity. DDA can be quantified and decomposed into species dark diversity affinity (dda_sp_) and site dark diversity affinity (dda_site_), allowing them to be separately associated with environmental characteristics and functional traits.

The dataset required for this method includes species occurrence data, location-specific suitability (probability values of the specific ecological suitability of each species at a particular location obtained by the hypergeometric distribution method), environmental characteristics of the site, and functional traits of the species. The environmental characteristics of the site include stand density (SDI), the total aboveground biomass (AGB), Elevation, Slope, soil pH (PH), soil water content (WCOS), the first and second principal components (PC1 and PC2) were generated through principal component analysis (PCA) of environmental variables related to soil nutrients and enzyme activity (see **Supplementary Figure 2**). functional traits of the species include Mycorrhizal type, seed mass (SM), stem density (WD), leaf nitrogen content (LTN), leaf phosphorus content (LTP), leaf organic carbon (LOC), leaf dry matter content (LDMC), specific leaf area (SLA) and leaf area (LA).

First, a unified species-site model should be constructed, which consisted of three operational components:

(1) Using the logistic regression model, estimates of dda_sp_ and dda_site_ were combined to form a unified DDA metric, see Equation 2:

(2) $$\text{logit}\left(\text{DD}{\text{A}}_{ij}\right)=\left(\mathrm{logit}\left(\text{dd}{\text{a}}_{sp-i}\right)+\text{logit}\left(\text{dd}{\text{a}}_{site-j}\right)\right)/2$$

where $DDA_{i,j}$ represents the DDA of species *i* at location *j*.

(2) The estimated DDA was used to adjust the suitability of the site (suit) to predict the probability of species occurrence (*p*). The adjusted occurrence probability was calculated using the following Equation 3:

(3) $$\text{logit}\left(p_{ij}\right)=\text{logit}\left(\left[1-\text{DD}{\text{A}}_{ij}\right]\times \mathrm{suit}{\text{t}}_{ij}\right)+\delta$$

where $p_{i,j}$ denotes the probability of occurrence of species *i* at location *j* and δ is a constant for adjusting the predicted occurrence probability to the observed occurrence rate level. The Equation 4 for obtaining δ is as follows:

(4) $$\delta =\text{logit}\left(\bar{prab}\right)-\text{logit}\left(\left[1-0.5\right]\times \bar{suit}\right)$$

(3) The observed species presence/absence data (*prab*) were linked to the predicted occurrence probability (*p*) if it obeys the Bernoulli distribution, thus allowing the inference of parameters (Lemoine, 2019).

We then applied Bayesian method to estimate the parameters of the species-site unified model: a, b, dda and DDA. To address the separation problem in logistic regression, we used weakly informative priors. Specifically, we used the Cauchy distribution and constrained the prior distribution to the center to maintain the conservativeness of the inference. The scale parameters for the prior distributions follow Gelman et al. (2008). All numeric predictor variables were standardized before model fitting (mean adjusted to 0, standard deviation adjusted to 0.5). The scale parameter for the intercept parameter a was set to 0.5. The scale parameter for the regression coefficient parameter b was set to 2.5. Bayesian MCMC sampling (an iterative algorithm) was performed using the Gibbs sampler JAGS 4.3.0, with a total of three MCMC chains 4000 iterating to adjust the DDA parameters until convergence (convergence was achieved with the Gelman-Rubin statistic R-hat ≤ 1.1 (Gelman and Rubin, 1992)), When the difference between the results of the three chains was very small and tended to stabilize, with at least 333 post-burn-in posterior samples having been retained for each chain, convergence was deemed satisfactory). Refer to Fujinuma and Pärtel (2023) for technical details. To assess the sensitivity of our model to MCMC configurations, we performed a robustness check on a randomly selected representative subset of the data. We compared the results of the initial protocol-based settings (3 chains, 4,000 iterations) against a more conservative configuration (4 independent chains without seed resetting, 20,000 burn-in, and 20,000 sampling iterations). This step was taken to ensure that the chosen iteration length was sufficient to capture the posterior distributions accurately.

## 3 Results

### 3.1 Dark diversity, observed diversity and CCI change patterns at different recovery stages

**Figure 2:**
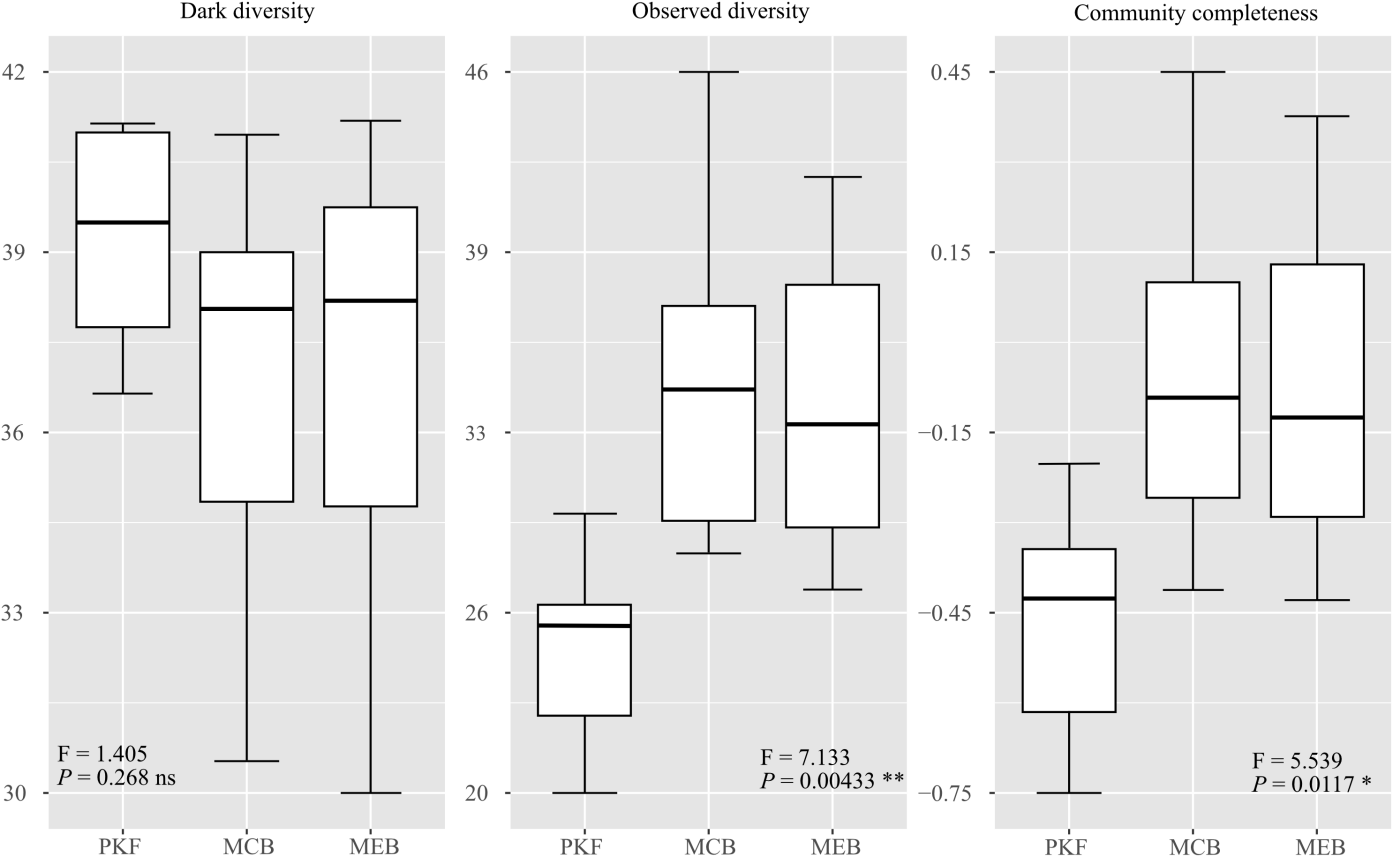
ANOVA results of dark diversity, observed diversity and community completeness at different natural recovery stages. Among them, dark diversity refers to site dark diversity, and community completeness refers to CCI. The box plot shows 1.5 times the median (line in the middle of the box) and the interquartile distance (box) ± interquartile distance (whisker).

The results showed (**Figure 2**; **Supplementary Table 2**) that the observed diversity significantly increased from PKF (25.4 ± 1.28) to MCB (35.2 ± 2.43) and from PKF to MEB (33.9 ± 1.94). However, the changes in community dark diversity from PKF (40.1 ± 0.61) to MCB (37.5 ± 1.43) and then to MEB (37.9 ± 1.30) were not significant (F = 1.405, p=0.268). This consistent high level of dark diversity across all stages suggested that despite the accumulation of observed species, a large and stable pool of potential colonizers remained excluded from the local community throughout the chronosequence. This also indicated that observed diversity alone was insufficient to assess natural recovery. CCI, as the ratio of observed diversity to dark diversity, was significantly correlated with species richness (**Supplementary Figure 3**) while also considering potential species in the recovery process. It rapidly increased significantly from PKF (-0.44 ± 0.06) to MCB (-0.07 ± 0.10) and then stabilizes at MEB (-0.12 ± 0.09), reflecting a transition in the recovery dynamics: an early phase of rapid stochastic reassembly followed by a later phase of more stringent, niche-based filtering. This change pattern allows us to successfully quantify the recovery process without the need for an external reference ecosystem.

### 3.2 Early warning species in different recovery stages

**Figure 3:**
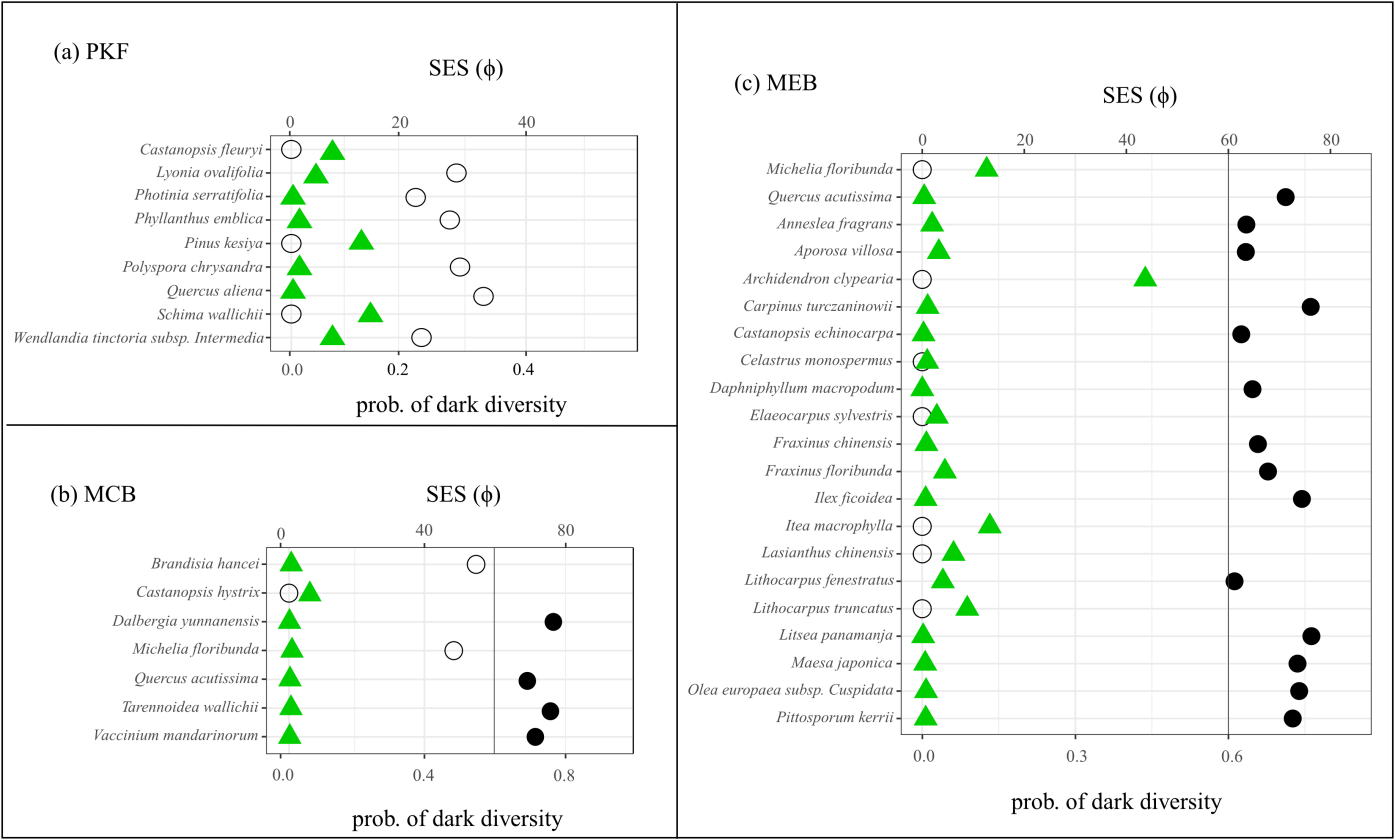
Relations between the standardized effect size of functional association (SES-ϕ) for diagnostic species based on functional traits (green triangles), and their average probability of dark diversity (circles) among each natural recovery stages (**(a)** PKF; **(b)** MCB; **(c)** MEB). The dark circles represent the species with a probability of dark diversity higher than 0.6.

A method based on species functional traits was used to detect diagnostic species and non-diagnostic species for three recovery stages. The results show that a different number of species had significant associations with the habitats of each recovery stage (p< 0.05). Among them, the number of diagnostic species for MEB (n=21) was the highest, while that for PKF (n=9) and MCB (n=7) was lower (**Figure 3**; **Supplementary Table 3**).

Combining the diagnostic species with the dark diversity of the species calculated above, we identified early warning species for each stage. There were no significant difference in the size of dark diversity between diagnostic species and non-diagnostic species during each recovery stage (*p* > 0.05; **Supplementary Figure 5**), supporting the method’s feasibility. In PKF, the dark diversity of all diagnostic species ranged from 0 to 0.4, with no early warning species. In MCB and MEB, a small portion of diagnostic species had dark diversity within the range of 0 to 0.4, while most species (4 in MCB and 14 in MEB) exceeded the 0.6 threshold. These species were all considered early warning species for the recovery stage, some light-loving transition species in the MCB, such as *Dalbergia yunnanensis*, *Quercus acutissima*, *Tarennoidea wallichii* and *Vaccinium mandarinorum*, as well as the shade-tolerant climax species in MEB, such as *Anneslea fragrans*, *Castanopsis echinocarpa* and *Lithocarpus fenestratus* in MEB. In summary, the identification of early-warning species provides a stage-specific diagnostic tool, these species-specific patterns are critical for direct management.

### 3.3 The relationship between dark diversity with functional traits and environmental characteristics at different recovery stages

The methodological rigor of our Bayesian decomposition is further supported by a sensitivity analysis conducted on a data subset. The comparison revealed (**Supplementary Figure 6**) that extending the MCMC chains from 4,000 to 20,000 iterations yielded virtually identical posterior modes and parameter signs. This consistency demonstrates that the 4,000-iteration length adopted from the Fujinuma and Pärtel (2023) protocol is sufficient for the model to reach satisfactory convergence in our study context, and that the reported ecological patterns are robust to MCMC sampling depth.

**Figure 4:**
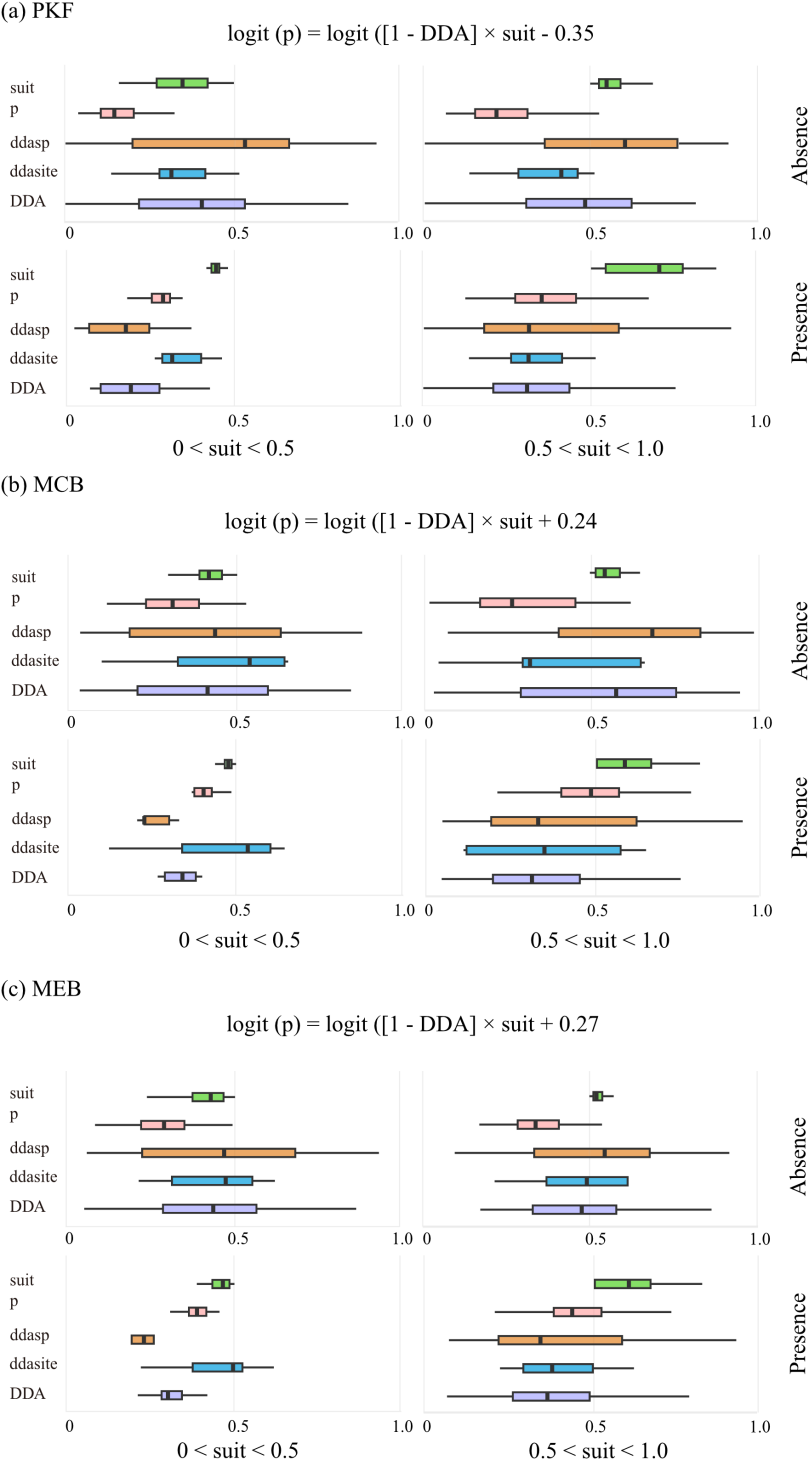
The estimated parameters of the three recovery stages (**(a)** PKF; **(b)** MCB; **(c)** MEB) are divided into four subsets based on site-specific suitabilities below/above 0.5 (left and right panels) and then by presence/absence (upper and lower panels). The distribution of estimated parameters in each subset is shown using box plots. The included model parameters are: p (presence likelihood), suit (suit value), DDA (uniform DDA), dda_sp_ (species DDA), and dda_site_ (site DDA). δ represent the constant parameters of each metacommunity. Each estimate is the median value of 999 Bayesian posterior samples.

Based on the specific indicators (early warning species) identified in different recovery stages, we further employed a DDA model to quantify the general assembly rules across the entire species pool. The posterior distributions of DDA model parameters are shown in **Figure 4**. Subsets where species were present generally had lower DDA than subsets where they were absent, confirming DDA as a valid proxy for dark diversity. The distribution of dda_sp_ (orange boxplot) shows a significant shift in position and a large variation. In contrast, the distribution of dda_site_ (blue boxplot) changes much less, with a less obvious shift in median and high overlap between boxplots. dda_sp_ contributes more to DDA than dda_site_, and DDA is mainly regulated by dda_sp_.

**Figure 5:**
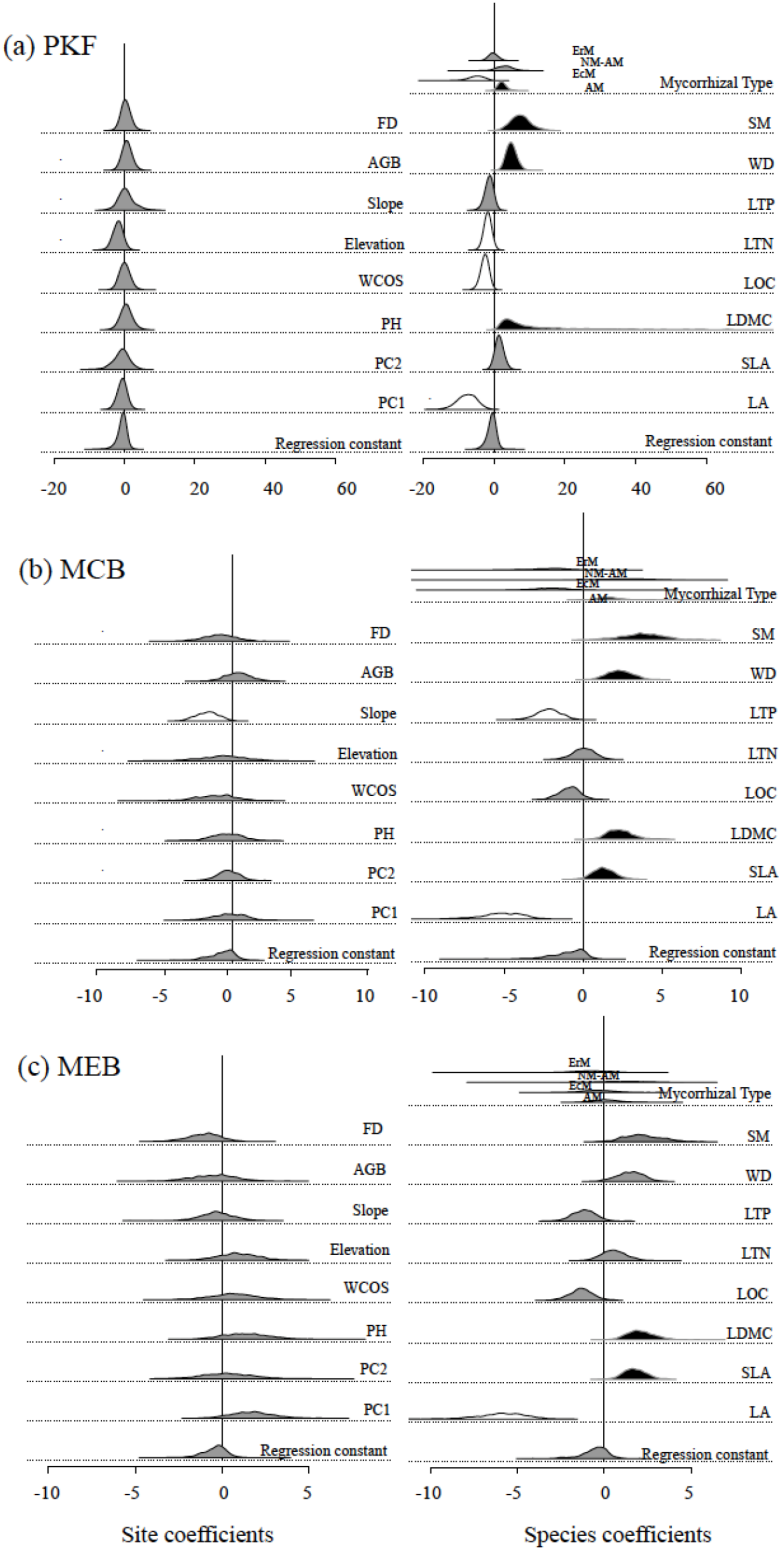
Posterior distributions of the logistic regression coefficients of dda_sp_ and dda_site_ in the model at different natural recovery stages (**(a)** PKF; **(b)** MCB; **(c)** MEB). The left and right columns show the estimated parameters of the species and the site explanatory variables, respectively. The density distribution is indicated using colors based on the 95% confidence interval (0.025–0.975): black denotes significantly positive (higher propensity for dark diversity, functional traits are significantly positively correlated with functional diversity), white denotes significantly negative (lower propensity for dark diversity, functional traits are significantly negative correlated with functional diversity), and gray denotes insignificant correlation (functional traits are not significantly correlated with dark diversity). Before model matching, all numerical explanatory variables were normalized to mean = 0 and standard deviation = 0.5.

At the site level (dda_site_), environmental characteristics exhibited almost no significant correlation with DDA across all successional stages, with the sole exception of a negative correlation with slope during the MCB stage (**Figure 5a**). This striking lack of abiotic influence suggested that habitat-level environmental filters were not the primary drivers of species exclusion in this system. At the species level (dda_sp_), the DDA model revealed a progressive shift in the types of functional traits influencing dark diversity as recovery proceeded (**Figure 5b**): In PKF, DDA was primarily associated with regeneration and wood-structure traits. Specifically, species with larger seed mass (SM) and higher stem wood density (WD) showed a significantly higher propensity to belong to the dark diversity (i.e., they were more likely to be missing). Additionally, mycorrhizal type played a role, with AM species being more likely and EcM species being less likely to be in the dark diversity. In MCB, the influence of seed mass persisted, but leaf-based physiological traits began to emerge as significant factors. For instance, specific leaf area (SLA) exhibited a significant positive correlation with ddasp, while leaf phosphorus content (LTP) showed a negative correlation. In MEB, the influence of traits related to initial colonization (e.g., seed mass and wood density) completely disappeared. Instead, dark diversity affinity was exclusively determined by resource acquisition traits, including leaf dry matter content (LDMC), SLA, and leaf area. In summary, the model results indicate a clear successional turnover in the functional filters of dark diversity, shifting from an emphasis on seed and wood traits in early stages to leaf and resource-related traits in the late stage.

## 4 Discussion

### 4.1 Measuring the success of forest natural recovery based on dark diversity

Our analysis of CCI dynamics across recovery stages and the association between CCI and species richness reveals its utility as a recovery metric. Although CCI is significantly correlated with species richness, as a relative index, CCI reflects the proportion of the actual species pool to the ecological potential of the area, providing a more detailed and specific perspective on restoration integrity. As Deschênes et al. (2024) pointed out, although CCI is expected to show a correlation with species richness as species accumulate, it considers the dynamic nature of the potential species pool, while simple richness counts ignore this. CCI is lowest in the early stage (PKF), indicating that although environmental conditions may allow its existence, many suitable species have not yet colonized. Significant increases in CCI during mid-recovery (MCB) align with the reappearance of “hidden” species from seed banks or surrounding areas as habitat structure and resources improve (Wilson et al., 2011; Lewis et al., 2017; Dong et al., 2019). During the late recovery (MEB) stage, there was a non-significant slight decline in CCI, which may reflect a shift in the driving factors of community assembly. In late-successional forests, stronger biological filtering (such as niche occupation by dominant late-successional species or intensified light competition under closed canopies) may limit additional species entering from the potential pool (Zhai et al., 2017; Huang et al., 2024). This trend of niche stability may lead to a higher proportion of dark diversity in mature communities, as environmental filters become more restrictive (Riibak et al., 2024). Overall, these trends indicate a gradual and significant elevation of CCI throughout the natural recovery process of forests; when CCI reaches a state of non-significant fluctuation, it can be inferred that the forest natural recovery is nearing completion. A notable additional finding emerged from our study: the decrease in dark diversity from the early to the late stage of natural recovery did not match the increase in observed diversity. This discrepancy arises because ongoing environmental amelioration during natural recovery attracts a broader array of species from more extensive surrounding areas, thereby expanding the size of the local potential species pool (Riibak et al., 2024; Wan and Wang, 2025). Therefore, even if observed diversity increased significantly during the recovery process, the corresponding dark diversity would not decrease significantly. This further indicates that CCI calculated based on dark diversity is a more superior indicator of community integrity compared to that calculated based on observed diversity (observed species richness/total species pool) (Pärtel et al., 2013). However, it is important to note that establishing a reference baseline based on a homogeneous environment is also crucial when using CCI to infer the natural recovery state, in order to eliminate the influence of environmental background noise.

In forest ecosystem restoration projects, assessing the natural recovery status of forests often requires an undisturbed ecosystem as a reference (Deschênes et al., 2024). Where ideal reference sites are unavailable, the CCI can serve as a new indicator to measure the success of forest natural recovery. We conduct continuous monitoring starting at a certain time during forest natural recovery, calculating and comparing the CCI at each time point. When the CCI continues to increase, it indicates that the forest is undergoing natural recovery, and this is also the optimal time for artificial intervention to accelerate natural recovery. When the CCI stops increasing significantly after a period of growth, it usually means that the natural recovery of the community has reached its optimal state and the community is becoming stable. At this point, if the restorer still feels that the recovery has not met the expected goals, this can serve as a new trigger to initiate active adjustment measures in restoration management (Moeslund et al., 2017; Deschênes et al., 2024).

### 4.2 Identification of early warning species based on dark diversity

Early warning species are related to the functional centers of their habitats, but often occupy ecological marginal positions (Dalle Fratte et al., 2022). Therefore, they can highlight the current status and existing problems of ecosystem natural recovery to some extent. The method of identifying early warning species by combining diagnostic species and species dark diversity has been successfully tested in some habitat types, such as alpine habitats (Dalle Fratte et al., 2022). In this study, we applied this method to a structurally complex forest ecosystem. The results showed that there were no early warning species in the early stage of natural forest recovery. At this stage, diagnostic species are typically pioneer species with high dispersal ability and strong environmental tolerance. Once the environment becomes suitable, they quickly occupy habitats, resulting in a very low probability of species absence (dark diversity), hence no early warning species. In the middle stage of natural recovery, early warning species included *Dalbergia yunnanensis*, *Quercus acutissima*, *Tarennoidea wallichii*, and *Vaccinium mandarinorum*. These species belong to shrubs or small trees, have a high specific leaf area (SLA), and are resource-acquisition type plants that mainly grow in high-light environments. When they become early warning species, it means that the functional center of the current stage of habitat has exceeded the ecological niche of these species. This absence is not a sign of failure to recover, but evidence of niche displacement, confirming that the plant community is successfully evolving towards a more compact canopy and a stable state, with species belonging to this strategy being selected out. Moreover, some studies also indicate that these species are transitional species in the monsoon evergreen broad-leaved forests (Tang, 2015). In the late stage of natural recovery, early warning species include species such as *Anneslea fragrans*, *Castanopsis echinocarpa* and *Lithocarpus fenestratus*. These species have a lower specific leaf area, belong to resource conservative plants, and mainly grow under closed canopies and moist, shaded forests. As representatives of the top-level monsoon evergreen broad-leaved forest communities, their reduction may indicate that the forest is beginning to regress from a stable, complex mature stage to an unstable, structurally simple early stage (Tang, 2015).

Ecosystems will continue to be affected by human activities and environmental changes during the process of natural recovery. We need to monitor the structure and function of the current ecosystem and early changes in environmental conditions in real time to promptly identify problems that may arise during natural recovery, thus gaining a critical time window for implementing intervention measures (such as reducing grazing intensity, restoring disturbance mechanisms). The framework adopted here combines functional diagnostic species with dark diversity, providing a repeatable and standardized method for ecosystem assessment. On one hand, by replacing qualitative expert intuition with algorithmic processes, especially the combination of functional trait clustering and statistical occupancy probability (Beals index), the subjectivity of species identification is reduced; on the other hand, the introduction of dark diversity can transform the originally overlooked potential species into critical ecological information, and can better lock in diagnostic species that have disappeared due to changes in environmental conditions, thereby effectively compensating for the limitations of traditional observation methods in sampling bias and time lag, and achieving a sensitive capture and prospective assessment of ecosystem degradation risks and functional space gaps (Kovač et al., 2016; Rodríguez-Rojo et al., 2020; Delbosc et al., 2021). Early warning species are related to the functional core of their environment as part of indicator species. On one hand, we identify these species during the restoration process. By analyzing their ecological characteristics (such as their specific requirements for humidity, soil nutrients, and pollinators), we can precisely detect problems in the natural restoration process. For example, if all early warning species are shade-tolerant plants and they are disappearing, it indicates that the canopy layer is too dense during natural restoration, leading to a mismatch in light conditions. On the other hand, if early warning species are the dominant species in the naturally restored community, it suggests that the ecosystem may be facing degradation. Identifying them allows us to promptly detect potential issues before the system fully degrades back to its original state, thus deciding whether to transition from passive natural restoration to human intervention.

### 4.3 Identifying obstacles to natural forest restoration based on dark diversity

Understanding why some species that should exist are missing in certain regions (dark diversity driving factors) can clarify the ecological barriers to species establishment, such as, dispersed limitations, disturbances or competition, etc. Fujinuma and Pärtel (2023) used various assemblages (from plants to mammals) representing different regions (from Central Europe to New Zealand, from North America East to South America Central) as meta-communities for analysis, confirming the hypothesis that the mechanisms shaping dark diversity operate at both species and site levels. However, our study results showed that that site-level environmental characteristics—including, forest structure, and topography—showed almost no significant relationship with DDA (**Figure 5a**). This result suggests that within the studied landscape, environment constraints are not the primary bottlenecks for species re-establishment. Instead, our findings indicate that dispersal limitations, biotic interactions, or historical contingencies play a much more decisive role in shaping community assembly than environmental factors. On one hand, high abiotic homogeneity in our study sites likely shifts the filtering pressure to biotic interactions; on the other hand, PCA-based compression of environmental information may overlook the influence of specific, fine-scale variables (e.g., micro-nutrients or micro-climate). Nevertheless, the overall lack of distinct environmental signals strongly indicates the dominance of species-level functional filtering processes.

Our study considers time dynamics while analyzing the drivers of dark diversity, allowing us to understand the factors hindering recovery in different stages of natural restoration. The early stage of natural recovery is the phase where plant communities successfully spread and rapidly establish. The results show that during this stages, seed mass, mycorrhizal type, stem density, and some leaf functional traits are all related to the formation of dark diversity. Specifically, species with larger seed are more likely to form dark diversity. Seed size usually reflects both the species’ dispersal ability and establishment ability (Myers and Harms, 2009). Although larger seeds are beneficial for the successful establishment of seedlings, they have lower production and weaker dispersal ability, making it difficult for them to spread over large areas to reach suitable regions for population establishment (Cain et al., 2000; Moles and Westoby, 2004; Riibak et al., 2015; Moeslund et al., 2017). Compared to other mycorrhizal types, AM species are more likely to form dark diversity, while EcM species are opposite. Mycorrhizal type is a key factor in the success of plant establishment. AM species have lower drought resistance and tolerance than EcM species, so they are less likely to successfully establish in a certain area (Krauss et al., 2004; Hempel et al., 2013). The relationship between stem density and dark diversity may reflect the species’ ability to adapt to environmental stress and niche differentiation. Plants with higher stem density usually grow more slowly, making them more likely to form dark diversity in the early and mid stages of natural recovery (Hodáňová, 1981). In addition, species with higher nutrient content have certain advantages during the establishment stage and are less likely to become part of dark diversity (Cornelissen et al., 2003; Moeslund et al., 2017). It is worth noting that species capable of rapidly acquiring resources (large leaf area, low leaf dry matter content) are less likely to become part of the hidden diversity during the early stages of natural recovery. Species with these characteristics are more likely to colonize the early successional habitats after disturbance (Gelman and Rubin, 1992). In the middle to late stages of natural recovery, the focus is more on the persistence of species in a given habitat. Once a species has spread to a certain location and successfully established itself, its competitive ability against other plants and tolerance to environmental changes determine whether it can survive long-term in that habitat. In the middle and late stages of natural recovery, the forest canopy gradually closes, reducing light intensity and intensifying competition among plants. At this time, species with low SLA are “resource conservers,” and they are less likely to become part of the dark diversity (Deschênes et al., 2024).

In restoration projects, identifying specific restoration barriers helps restorers develop targeted interventions to alleviate these obstacles and improve the success of the restoration project, rather than blindly carrying out large-scale vegetation reconstruction (Moeslund et al., 2017; Deschênes et al., 2024). Based on the results, we mainly propose the following general recommendations: (1) Dispersal limitation is a key factor for most species to become part of the habitat’s hidden diversity, and promoting species return through artificial assistance in dispersal can enhance biodiversity, provided that the introduced species conform to the natural distribution patterns of the local ecosystem; (2) Mycorrhizal fungi are important for the establishment and long-term survival of plant species, and inoculating certain mycorrhizal fungi can play a certain role in the successful restoration of plant communities; (3) A plant’s ability to acquire resources to some extent determines its competitive ability within the community. To ensure the successful establishment and survival of important species in resource-scarce habitats, it is necessary to artificially create suitable conditions based on the characteristics of the plants themselves. In conclusion, forest management should also focus on facilitating migration, creating dispersal corridors, or protecting animal dispersers to promote the arrival of these “functionally suitable but absent” species, rather than solely focusing on abiotic habitat modification.

### 4.4 Limitations of assessing forest natural recovery based on dark diversity

Our research integrates dark diversity, functional traits, and diagnostic organisms, providing a powerful tool for assessing the natural recovery of forest ecosystems. However, it must be acknowledged that some limitations exist to give the research findings contextual significance, which can be categorized into study-specific design limitations and the inherent methodological challenges of dark diversity methods. Regarding the study execution, the use of a chronosequence (space-for-time substitution) approach carries inherent assumptions. While we selected sites with similar parent materials and climates, the potential for site-specific history and environmental heterogeneity to influence successional trajectories cannot be entirely ruled out. Furthermore, our analysis relied on species-averaged trait values obtained from databases or literature, thereby ignoring trait variation. Given that species can exhibit significant plastic responses to local environmental changes during forest recovery, Ignore traits variations may lead to an underestimation of the adaptive capacity of certain species. Beyond these design constraints, the conceptual application of dark diversity presents inherent methodological challenges, particularly in the definition of the habitat species pool. The accuracy of dark diversity estimations depends heavily on the quality and scope of the regional species pool. As Deschênes et al. (2024) pointed out, choosing a reference target is increasingly difficult under global change, where historical baselines may no longer be realistic. Furthermore, using probability values to quantify dark diversity at the species level has certain logical ambiguities. For example, a species may have a lower dark diversity because it is a common species already present in the sample plot, or because it is a rare species not accepted by the environment. This can lead to overlap of species in the probability distribution. In conclusion, while these limitations exist, our framework serves as a proactive diagnostic tool. Future research should aim to incorporate field trait measurements and long-term monitoring data, and further study should be conducted to refine the probabilistic dark diversity method for practical application.

## 5 Conclusion

This study developed and applied an integrated dark diversity framework to assess forest natural recovery across different stages. We demonstrated that the Community Completeness Index (CCI) considers the dynamic characteristics of the potential species pool, providing a more comprehensive and detailed measure of recovery success. Early warning species for different recovery stages were identified through the cross recognition of diagnostic species and hidden diversity, allowing us to predict potential issues in the natural recovery process and intervene in a timely manner. The main obstacles to natural recovery shift from dispersal and establishment limitations in early/mid stages (related to seed mass, mycorrhizal types, and wood density) to competitive interactions in later stages (related to resource acquisition traits). Crucially, we found that species traits were a stronger driver of absence than local environment conditions, highlighting dispersal and biotic interactions as primary restoration targets. While based on a single regional chronosequence, our framework offers a replicable, mechanism-based approach to diagnose recovery barriers. For practitioners, it argues for precision restoration—shifting from broad-scale planting to targeted strategies like assisted dispersal or mycorrhizal inoculation in early stages, and canopy management in later stages. Future work should validate this framework across diverse ecosystems and integrate it with long-term monitoring to test the efficacy of interventions designed to reduce dark diversity.

## Data Availability

The original contributions presented in the study are included in the article/**Supplementary Material**. Further inquiries can be directed to the corresponding author.

## References

[B1] Atkinson J. Bonser S. P. (2020). Active” and “passive” ecological restoration strategies in meta-analysis. Restor. Ecol. 28, 1032–1035. doi: 10.1111/rec.13229, PMID: 41778641

[B2] Aubin I. Deschênes É. Santala K. R. Emilson E. J. S. Schoonmaker A. L. McIntosh A. C. S. . (2024). Restoring forest ecosystem services through trait-based ecology. Environ. Rev. 32, 498–524. doi: 10.1139/er-2023-0130, PMID: 36563491

[B3] Boussarie G. Bakker J. Wangensteen O. S. Mariani S. Bonnin L. Juhel J.-B. . (2018). Environmental DNA illuminates the dark diversity of sharks. Sci. Adv. 4, eaap9661. doi: 10.1126/sciadv.aap9661, PMID: 29732403 PMC5931749

[B4] Brockerhoff E. G. Barbaro L. Castagneyrol B. Forrester D. I. Gardiner B. González-Olabarria J. R. . (2017). Forest biodiversity, ecosystem functioning and the provision of ecosystem services. Biodivers. Conserv. 26, 3005–3035. doi: 10.1007/s10531-017-1453-2, PMID: 41776007

[B5] Cain M. L. Milligan B. G. Strand A. E. (2000). Long-distance seed dispersal in plant populations. Am. J. Bot. 87, 1217–1227. doi: 10.2307/2656714 10991892

[B6] Cam E. Nichols J. D. Sauer J. R. Hines J. E. Flather C. H. (2000). Relative species richness and community completeness: birds and urbanization in the mid-atlantic states. Ecol. Appl. 10, 1196–1210. doi: 10.1890/1051-0761(2000)010[1196:RSRACC]2.0.CO;2

[B7] Carlucci M. B. Brancalion P. H. S. Rodrigues R. R. Loyola R. Cianciaruso M. V. (2020). Functional traits and ecosystem services in ecological restoration. Restor. Ecol. 28, 1372–1383. doi: 10.1111/rec.13279, PMID: 41778641

[B8] Carmona C. P. Pärtel M. (2021). Estimating probabilistic site-specific species pools and dark diversity from co-occurrence data. Glob. Ecol. Biogeogr. 30, 316–326. doi: 10.1111/geb.13203, PMID: 41778641

[B9] Caro T. M. Girling S. (2010). Conservation by proxy: indicator, umbrella, keystone, flagship, and other surrogate species (Washington, DC: Island Press).

[B10] Chen S. Chen J. Jiang C. Yao R. T. Xue J. Bai Y. . (2022). Trends in research on forest ecosystem services in the most recent 20 years: A bibliometric analysis. Forests 13, 1087. doi: 10.3390/f13071087, PMID: 41725453

[B11] Chollet S. Dano M. Thiébaut G. Jung V. (2025). Dark diversity and habitat conservation status: Two sides of the same coin for conservation and restoration? Ecol. Indic. 170, 112990. doi: 10.1016/j.ecolind.2024.112990, PMID: 41777705

[B12] Condit R. (1995). Research in large, long-term tropical forest plots. Trends Ecol. Evol. 10, 18–22. doi: 10.1016/S0169-5347(00)88955-7, PMID: 21236939

[B13] Cornelissen J. H. C. Lavorel S. Garnier E. Díaz S. Buchmann N. Gurvich D. E. . (2003). A handbook of protocols for standardised and easy measurement of plant functional traits worldwide. Aust. J. Bot. 51, 335. doi: 10.1071/BT02124, PMID: 41161682

[B14] Dalle Fratte M. Caccianiga M. Ricotta C. Cerabolini B. E. L. (2022). Identifying typical and early warning species by the combination of functional-based diagnostic species and dark diversity. Biodivers. Conserv. 31, 1735–1753. doi: 10.1007/s10531-022-02427-4, PMID: 41776007

[B15] De Bello F. Fibich P. Zelený D. Kopecký M. Mudrák O. Chytrý M. . (2016). Measuring size and composition of species pools: a comparison of dark diversity estimates. Ecol. Evol. 6, 4088–4101. doi: 10.1002/ece3.2169, PMID: 27516866 PMC4877358

[B16] De Bello F. Price J. N. Münkemüller T. Liira J. Zobel M. Thuiller W. . (2012). Functional species pool framework to test for biotic effects on community assembly. Ecology 93, 2263–2273. doi: 10.1890/11-1394.1, PMID: 23185887

[B17] DeForest J. L. (2009). The influence of time, storage temperature, and substrate age on potential soil enzyme activity in acidic forest soils using MUB-linked substrates and l-DOPA. Soil Biol. Biochem. 41, 1180–1186. doi: 10.1016/j.soilbio.2009.02.029, PMID: 41777705

[B18] Delbosc P. Lagrange I. Rozo C. Bensettiti F. Bouzillé J.-B. Evans D. . (2021). Assessing the conservation status of coastal habitats under Article 17 of the EU Habitats Directive. Biol. Conserv. 254, 108935. doi: 10.1016/j.biocon.2020.108935, PMID: 41777705

[B19] Deschênes É. Santala K. R. Lavigne J. Aubin I. (2024). Using a trait-based dark diversity approach to evaluate natural recovery potential in forests. Restor. Ecol. 32, e14251. doi: 10.1111/rec.14251, PMID: 41778641

[B20] Dong K. Hao G. Yang N. Zhang J. Ding X. Ren H. . (2019). Community assembly mechanisms and succession processes significantly differ among treatments during the restoration of Stipa grandis – Leymus chinensis communities. Sci. Rep. 9, 16289. doi: 10.1038/s41598-019-52734-0, PMID: 31705024 PMC6841928

[B21] Feng X. Huang H. Wang Y. Li L. Tian Y. (2025). Integrating dark diversity perspective into species conservation: A novel regional species protection index (RSPI) for identifying priority areas. Ecol. Indic. 179, 114173. doi: 10.1016/j.ecolind.2025.114173, PMID: 41777705

[B22] Fujinuma J. Pärtel M. (2023). Decomposing dark diversity affinities of species and sites using Bayesian method: What accounts for absences of species at suitable sites? Methods Ecol. Evol. 14, 1796–1807. doi: 10.1111/2041-210X.14109, PMID: 41778641

[B23] Gatica-Saavedra P. Echeverría C. Nelson C. R. (2017). Ecological indicators for assessing ecological success of forest restoration: a world review. Restor. Ecol. 25, 850–857. doi: 10.1111/rec.12586, PMID: 41778641

[B24] Gelman A. Jakulin A. Pittau M. G. Su Y . (2008). A weakly informative default prior distribution for logistic and other regression model. Ann. Appl. Stat. 2, 1360–1383. doi: 10.1214/08-AOAS191, PMID: 41780105

[B25] Gelman A. Rubin D. B. (1992). Inference from iterative simulation using multiple sequences. Stat. Sci. 7, 457–447. doi: 10.1214/ss/1177011136

[B26] Helm A. Zobel M. Moles A. T. Szava-Kovats R. Pärtel M. (2015). Characteristic and derived diversity: implementing the species pool concept to quantify conservation condition of habitats. Divers. Distrib. 21, 711–721. doi: 10.1111/ddi.12285, PMID: 41778641

[B27] Hempel S. Götzenberger L. Kühn I. Michalski S. G. Rillig M. C. Zobel M. . (2013). Mycorrhizas in the Central European flora: relationships with plant life history traits and ecology. Ecology 94, 1389–1399. doi: 10.1890/12-1700.1, PMID: 23923502

[B28] Hemrová L. Münzbergová Z. (2015). The effects of plant traits on species’ responses to present and historical patch configurations and patch age. Oikos 124, 437–445. doi: 10.1111/oik.01130, PMID: 41778641

[B29] Hodáňová D. (1981). Plant strategies and vegetation processes Vol. 23. Ed. Grime J. P. (Chichester-New York-Brisbane-Toronto: John Wiley & Sons, Ltd.), 254–254. doi: 10.1007/BF02895358, PMID:

[B30] Hosseini S. Amirnejad H. Azadi H. (2024). Impacts of Hyrcanian forest ecosystem loss: the case of Northern Iran. Environ. Dev. Sustain. 27, 14397–14418. doi: 10.1007/s10668-023-04408-1, PMID: 41776007

[B31] Hostens L. Van Meerbeek K. Wiegmans D. Larson K. Lenoir J. Clavel J. . (2023). The drivers of dark diversity in the Scandinavian mountains are metric-dependent. J. Veg. Sci. 34, e13212. doi: 10.1111/jvs.13212, PMID: 41778641

[B32] Huang L.-M. Xu H.-Q. Yu J.-Y. Chen Y.-H. Wang J.-Q. Ji F.-F. . (2024). The ecological niches and interspecific associations of the dominant fishes in the Xiamen seas, China. Fishes 9, 354. doi: 10.3390/fishes9090354, PMID: 41725453

[B33] Kattge J. Díaz S. Lavorel S. Prentice I. C. Leadley P. Bönisch G. . (2011). TRY – a global database of plant traits. Glob. Change Biol. 17, 2905–2935. doi: 10.1111/j.1365-2486.2011.02451.x, PMID: 41778641

[B34] Kleyer M. Bekker R. M. Knevel I. C. Bakker J. P. Thompson K. Sonnenschein M. . (2008). The LEDA Traitbase: a database of life-history traits of the Northwest European flora. J. Ecol. 96, 1266–1274. doi: 10.1111/j.1365-2745.2008.01430.x, PMID: 41778641

[B35] Kovač M. Kutnar L. Hladnik D. (2016). Assessing biodiversity and conservation status of the Natura 2000 forest habitat types: Tools for designated forestlands stewardship. For. Ecol. Manage. 359, 256–267. doi: 10.1016/j.foreco.2015.10.011, PMID: 41777705

[B36] Krauss J. Klein A.-M. Steffan-Dewenter I. Tscharntke T. (2004). Effects of habitat area, isolation, and landscape diversity on plant species richness of calcareous grasslands. Biodivers. Conserv. 13, 1427–1439. doi: 10.1023/B:BIOC.0000021323.18165.58, PMID: 38124636

[B37] Lemoine N. P. (2019). Moving beyond noninformative priors: why and how to choose weakly informative priors in Bayesian analyses. Oikos 128, 912–928. doi: 10.1111/oik.05985, PMID: 41778641

[B38] Lewis R. J. De Bello F. Bennett J. A. Fibich P. Finerty G. E. Götzenberger L. . (2017). Applying the dark diversity concept to nature conservation. Conserv. Biol. 31, 40–47. doi: 10.1111/cobi.12723, PMID: 27027266

[B39] Li J. Prentice I. C. (2024). Global patterns of plant functional traits and their relationships to climate. Commun. Biol. 7, 1136. doi: 10.1038/s42003-024-06777-3, PMID: 39271947 PMC11399309

[B40] Liu U. Cossu T. A. Dickie J. (2019). Royal Botanic Gardens, Kew’s Seed Information Database (SID): A compilation of taxon-based biological seed characteristics or traits. Biodivers. Inf. Sci. Stand. 3, e37030. doi: 10.3897/biss.3.37030, PMID: 41658337

[B41] Liu Q. Wang S. Ma R. Huang F. Li J. Ye S. . (2024). Comparative analysis of forest soil carbon sink and source based on bibliometrics: Development, hotspots, and trends. J. Clean. Prod. 480, 144106. doi: 10.1016/j.jclepro.2024.144106, PMID: 41777705

[B42] Moeslund J. E. Brunbjerg A. K. Clausen K. K. Dalby L. Fløjgaard C. Juel A. . (2017). Using dark diversity and plant characteristics to guide conservation and restoration. J. Appl. Ecol. 54, 1730–1741. doi: 10.1111/1365-2664.12867, PMID: 41778641

[B43] Moles A. T. Westoby M. (2004). Seedling survival and seed size: a synthesis of the literature. J. Ecol. 92, 372–383. doi: 10.1111/j.0022-0477.2004.00884.x, PMID: 41778641

[B44] Myers J. A. Harms K. E. (2009). Seed arrival, ecological filters, and plant species richness: a meta-analysis. Ecol. Lett. 12, 1250–1260. doi: 10.1111/j.1461-0248.2009.01373.x, PMID: 19723285

[B45] Nicod C. Leys B. Ferrez Y. Manneville V. Mouly A. Greffier B. . (2019). Towards the assessment of biodiversity and management practices in mountain pastures using diagnostic species? Ecol. Indic. 107, 105584. doi: 10.1016/j.ecolind.2019.105584, PMID: 41777705

[B46] Paganeli B. Fujinuma J. Trindade D. P. F. Carmona C. P. Pärtel M. (2024). A roadmap to carefully select methods for dark-diversity studies. J. Veg. Sci. 35, e13264. doi: 10.1111/jvs.13264, PMID: 41778641

[B47] Pärtel M. Szava-Kovats R. Zobel M. (2011). Dark diversity: shedding light on absent species. Trends Ecol. Evol. 26, 124–128. doi: 10.1016/j.tree.2010.12.004, PMID: 21195505

[B48] Pärtel M. Szava-Kovats R. Zobel M. (2013). Community completeness: linking local and dark diversity within the species pool concept. Folia Geobot. 48, 307–317. doi: 10.1007/s12224-013-9169-x, PMID: 41776007

[B49] Qin Y. Xiao X. Wigneron J.-P. Ciais P. Canadell J. G. Brandt M. (2022). Large loss and rapid recovery of vegetation cover and aboveground biomass over forest areas in Australia during 2019–2020. Remote Sens. Environ. 278, 113087. doi: 10.1016/j.rse.2022.113087, PMID: 41777705

[B50] Ricotta C. Acosta A. T. R. Caccianiga M. Cerabolini B. E. L. Godefroid S. Carboni M. (2020). From abundance-based to functional-based indicator species. Ecol. Indic. 118, 106761. doi: 10.1016/j.ecolind.2020.106761, PMID: 41777705

[B51] Riedl M. Němec M. Jarský V. (2024). Thirty years of research on ecosystem services: the socio-economic role of forest visits and foraging in enhancing human well-being. Forests 15, 1845. doi: 10.3390/f15111845, PMID: 41725453

[B52] Riibak K. Noreika N. Helm A. Öpik M. Kook E. Kasari-Toussaint L. (2024). Plants, fungi, and carabid beetles in temperate forests: both observed and dark diversity depend on habitat availability in space and time. Landsc. Ecol. 39, 158. doi: 10.1007/s10980-024-01960-7, PMID: 41776007

[B53] Riibak K. Reitalu T. Tamme R. Helm A. Gerhold P. Znamenskiy S. (2015). Dark diversity in dry calcareous grasslands is determined by dispersal ability and stress-tolerance. Ecography 38, 713–721. doi: 10.1111/ecog.01312, PMID: 41778641

[B54] Rodríguez-Rojo M. P. Font X. García-Mijangos I. Crespo G. Fernández-González F. (2020). An expert system as an applied tool for the conservation of semi-natural grasslands on the Iberian Peninsula. Biodivers. Conserv. 29, 1977–1992. doi: 10.1007/s10531-020-01963-1, PMID: 41776007

[B55] Shackelford N. Hobbs R. J. Burgar J. M. Erickson T. E. Fontaine J. B. (2013). Primed for change: developing ecological restoration for the 21st century. Restor. Ecol. 21, 297–304. doi: 10.1111/rec.12012, PMID: 41778641

[B56] Shang R. Li S. Huang X. Liu W. Lang X. Su J. (2021). Effects of soil properties and plant diversity on soil microbial community composition and diversity during secondary succession. Forests 12, 805. doi: 10.3390/f12060805, PMID: 41725453

[B57] Shang R. Li S. Huang X. Liu W. Lang X. Xu C. . (2023). Soil bacterial community and ecosystem multifunctionality regulated by keystone plant species during secondary succession. Land Degrad. Dev. 34, 5997–6008. doi: 10.1002/ldr.4892, PMID: 41778520

[B58] Soudzilovskaia N. A. Vaessen S. Barcelo M. He J. Rahimlou S. Abarenkov K. . (2020). FungalRoot: global online database of plant mycorrhizal associations. New Phytol. 227, 955–966. doi: 10.1111/nph.16569, PMID: 32239516

[B59] Tang C. Q. (2010). Subtropical montane evergreen broad-leaved forests of Yunnan, China: diversity, succession dynamics, human influence. Front. Earth Sci. China 4, 22–32. doi: 10.1007/s11707-009-0057-x, PMID: 41776007

[B60] Tang C. Q. (2015). The Subtropical Vegetation of Southwestern China: Plant Distribution, Diversity and Ecology, Plant and Vegetation (Dordrecht: Springer). doi: 10.1007/978-94-017-9741-2, PMID:

[B61] Tang L. Li R. Wang W. Li B. (2023). The probabilistic site-specific species pool and dark diversity in the terrestrialized urban mangroves. Ecol. Indic. 148, 110134. doi: 10.1016/j.ecolind.2023.110134, PMID: 41777705

[B62] Violle C. Navas M. Vile D. Kazakou E. Fortunel C. Hummel I. (2007). Let the concept of trait be functional! Oikos 116, 882–892. doi: 10.1111/j.0030-1299.2007.15559.x, PMID: 41778641

[B63] Wan J. Wang X. (2025). Environmental heterogeneity drives the dark diversity of global plant communities. J. Biogeogr. 52, e70043. doi: 10.1111/jbi.70043, PMID: 41778641

[B64] Wilson K. A. Lulow M. Burger J. Fang Y.-C. Andersen C. Olson D. . (2011). Optimal restoration: accounting for space, time and uncertainty: Prioritizing ecological restoration. J. Appl. Ecol. 48, 715–725. doi: 10.1111/j.1365-2664.2011.01975.x, PMID: 41778641

[B65] Zanzottera M. Dalle Fratte M. Caccianiga M. Pierce S. Cerabolini B. E. L. (2020). Community-level variation in plant functional traits and ecological strategies shapes habitat structure along succession gradients in alpine environment. Community Ecol. 21, 55–65. doi: 10.1007/s42974-020-00012-9, PMID: 41776007

[B66] Zhai D. Xu J. Dai Z. Schmidt-Vogt D. (2017). Lost in transition: Forest transition and natural forest loss in tropical China. Plant Divers. 39, 149–153. doi: 10.1016/j.pld.2017.05.005, PMID: 30159505 PMC6112266

[B67] Zhang R. Li S. Huang X. Li C. Xu C. Su J. (2024b). Diversity-biomass relationships are shaped by tree mycorrhizal associations and stand structural diversity at different spatial scales. For. Ecosyst. 11, 100234. doi: 10.1016/j.fecs.2024.100234, PMID: 41777705

[B68] Zhang X. Yin G. Ma Y. Fan J. Zhou J. (2024a). Comparison between artificial restoration and natural recovery in vegetation regrowth following high-frequency fire disturbances in the Hengduan Mountains, Southwest China. Ecol. Indic. 167, 112692. doi: 10.1016/j.ecolind.2024.112692, PMID: 41777705

[B69] Zhao G. Xu E. Yi X. Guo Y. Zhang K. (2023). Comparison of forest restorations with different burning severities using various restoration methods at tuqiang forestry bureau of greater Hinggan Mountains. Remote Sens. 15, 2683. doi: 10.3390/rs15102683, PMID: 41725453

