# Supplementary material for: Integrating dark diversity, functional traits, and diagnostic species: a framework to diagnose bottlenecks in forest recovery
Source: Front Plant Sci. 2026 Mar 10;17:1723617. doi: 10.3389/fpls.2026.1723617 (PMC13011820; doi:10.3389/fpls.2026.1723617)
Supplement: Supplementary file 1 [file Table1.docx]

Supplementary Material

# Supplementary

# Supplementary Tables

**Supplementary Table 1.** Basic characteristics of the sampling stands in different recovery stages.

| **Basic characteristics of plots** | **Types** | | |
| --- | --- | --- | --- |
|  | **PKF** | **MCB** | **MEB** |
| **Principal species of trees** | *Pinus kesiya*, *Castanopsis echinocarpa*, *Schima wallichii*, *Castanopsis fleuryi* | *Pinus kesiya*, *Castanopsis echinocarpa*, *Schima wallichii*, *Aporosa villosa* | *Castanopsis echinocarpa*, *Lithocarpus fenestratus, Anneslea fragrans*, *Machilus nanmu* |
| **Plot number** | 8 | 8 | 8 |
| **Woody plants number** | 276±19 | 256±13 | 316±13 |
| **Average elevation** | 1407 | 1372 | 1498 |
| **Mean gradient** | 17 | 14 | 11 |
| **Tree cover** | 74 | 80 | 86 |

**Supplementary Table 2.** The results of Tukey HSD post-hoc test.

| **group** | **stage1** | **stage2** | **estimate** | **conf.low** | **conf.high** | **p.adj** | **p.adj.signif** |
| --- | --- | --- | --- | --- | --- | --- | --- |
| Dark diversity | PKF | MCB | -2.577085 | -6.756328 | 1.602158 | 0.287 | ns |
|  | PKF | MEB | -2.189064 | -6.368307 | 1.990179 | 0.400 | ns |
|  | MCB | MEB | 0.388021 | -3.791222 | 4.567264 | 0.970 | ns |
| Observed diversity | PKF | MCB | 9.625 | 2.677084 | 16.572906 | 0.0059 | ** |
|  | PKF | MEB | 8.250 | 1.302084 | 15.197916 | 0.0182 | * |
|  | MCB | MEB | -1.375 | -8.322916 | 5.57296 | 0.8730 | ns |
| Community completeness | PKF | MCB | 0.38341959 | 0.06661538 | 0.7002238 | 0.016 | * |
|  | PKF | MEB | 0.33662353 | 0.01981932 | 0.6534277 | 0.036 | * |
|  | MCB | MEB | -0.04679607 | -0.36360028 | 0.2700081 | 0.927 | ns |

**Supplementary Table 3.** Calculation results for the functional associations (φ) based on functional characteristics (φ_traits).

| **sp** | **obs.MCB** | **rand.MCB** | **p.MCB** | **obs.MEB** | **rand.MEB** | **p.MEB** | **obs.PKF** | **rand.PKF** | **p.PKF** |
| --- | --- | --- | --- | --- | --- | --- | --- | --- | --- |
| *Adinandra.megaphylla* | 0 | 0.06868 | 1 | 0.172135 | 0.053901 | 0.1 | 0 | 0.049554 | 1 |
| *Adinandra.nitida* | 0 | 0.19711 | 1 | 0.560745 | 0.198243 | 0.15 | 0 | 0.165391 | 1 |
| *Albizia.julibrissin* | 0 | 0.1715 | 1 | 0.088239 | 0.242589 | 0.56 | 0.545664 | 0.219815 | 0.37 |
| *Anneslea.fragrans* | 10.38806 | 12.59489 | 0.79 | 16.99207 | 12.62692 | 0.02 | 10.41294 | 12.57127 | 0.84 |
| *Annona.squamosa* | 0 | 0.423873 | 1 | 1.101665 | 0.38442 | 0.01 | 0 | 0.293373 | 1 |
| *Apodytes.dimidiata* | 0.295968 | 0.135412 | 0.17 | 0 | 0.131511 | 1 | 0.101471 | 0.130515 | 0.61 |
| *Aporosa.villosa* | 19.37193 | 11.79481 | 0.07 | 2.902792 | 12.16174 | 1 | 13.80664 | 12.12481 | 0.41 |
| *Archidendron.clypearia* | 1.012065 | 0.724049 | 0.11 | 0.735651 | 0.624139 | 0.32 | 0.167365 | 0.566893 | 0.95 |
| *Ardisia.japonica* | 0 | 0.0438 | 1 | 0.127535 | 0.039935 | 0.32 | 0 | 0.0438 | 1 |
| *Ardisia.quinquegona* | 0 | 0.140468 | 1 | 0.421405 | 0.166008 | 0.4 | 0 | 0.114929 | 1 |
| *Betula.alnoides* | 1.158597 | 2.452119 | 0.81 | 4.956736 | 1.946144 | 0.01 | 0.426624 | 2.143694 | 0.93 |
| *Brandisia.hancei* | 1.017531 | 0.388657 | 0.01 | 0 | 0.372276 | 1 | 0.102327 | 0.358926 | 0.76 |
| *Carpinus.turczaninowii* | 5.526953 | 1.915904 | 0.06 | 0 | 1.73095 | 1 | 0.115801 | 1.995901 | 0.83 |
| *Castanopsis.calathiformis* | 1.537631 | 4.212008 | 0.85 | 9.755777 | 3.251243 | 0.01 | 0.106913 | 3.93707 | 0.99 |
| *Castanopsis.echinocarpa* | 44.51995 | 45.02387 | 0.48 | 67.9905 | 43.71098 | 0.01 | 19.49627 | 43.27187 | 1 |
| *Castanopsis.fleuryi* | 4.790598 | 7.802248 | 0.73 | 0 | 8.731713 | 1 | 19.92745 | 8.184088 | 0.01 |
| *Castanopsis.hystrix* | 10.57054 | 5.153533 | 0.01 | 1.427951 | 4.884073 | 0.99 | 3.269105 | 5.229991 | 0.89 |
| *Castanopsis.wattii* | 0 | 0.044135 | 1 | 0.114982 | 0.038327 | 0.34 | 0 | 0.03252 | 1 |
| *Celastrus.monospermus* | 0.397087 | 1.064419 | 0.92 | 2.684772 | 1.064494 | 0.01 | 0 | 0.952946 | 1 |
| *Choerospondias.axillaris* | 0.039862 | 0.016911 | 0.43 | 0 | 0.012885 | 1 | 0 | 0.010066 | 1 |
| *Cinnamomum.bejolghota* | 0 | 0.253568 | 1 | 0.750951 | 0.257902 | 0.01 | 0 | 0.239481 | 1 |
| *Cornus.controversa* | 0 | 0.148824 | 1 | 0.409266 | 0.134355 | 0.11 | 0 | 0.126087 | 1 |
| *Craibiodendron.stellatum* | 0.565028 | 0.452957 | 0.35 | 0 | 0.544367 | 1 | 0.930166 | 0.49787 | 0.15 |
| *Craspedolobium.unijugum* | 1.056613 | 1.042762 | 0.47 | 2.023905 | 0.977353 | 0.02 | 0 | 1.060403 | 1 |
| *Cyclobalanopsis.tomentosinervis* | 0.310202 | 0.707392 | 0.59 | 0 | 0.768886 | 1 | 1.855785 | 0.689709 | 0.1 |
| *Dalbergia.cultrata* | 0.214726 | 0.152442 | 0.36 | 0 | 0.142701 | 1 | 0.213417 | 0.133001 | 0.39 |
| *Dalbergia.stipulacea* | 0.559373 | 0.310053 | 0.08 | 0.078809 | 0.310167 | 0.92 | 0.239012 | 0.256975 | 0.55 |
| *Dalbergia.yunnanensis* | 0.22388 | 0.087442 | 0.03 | 0 | 0.067089 | 1 | 0 | 0.06935 | 1 |
| *Daphniphyllum.macropodum* | 0.036677 | 0.012967 | 0.36 | 0 | 0.009632 | 1 | 0 | 0.014078 | 1 |
| *Decaspermum.parviflorum* | 0.440769 | 0.687906 | 0.66 | 0.881348 | 0.764218 | 0.49 | 0.901757 | 0.771751 | 0.37 |
| *Dichotomanthes.tristaniicarpa* | 0.111812 | 0.250022 | 0.52 | 0 | 0.240987 | 1 | 0.647718 | 0.268521 | 0.36 |
| *Diospyros.kaki.var..silvestris* | 0.511984 | 0.456182 | 0.39 | 0.304231 | 0.426384 | 0.76 | 0.51565 | 0.449301 | 0.27 |
| *Elaeocarpus.balansae* | 0.005569 | 0.012474 | 0.86 | 0.03161 | 0.01287 | 0.01 | 0 | 0.011835 | 1 |
| *Elaeocarpus.decipiens* | 0 | 0.063516 | 1 | 0.209603 | 0.069868 | 0.34 | 0 | 0.076219 | 1 |
| *Elaeocarpus.sylvestris* | 0.522022 | 0.574378 | 0.46 | 0.921834 | 0.555538 | 0.15 | 0.21461 | 0.528549 | 0.85 |
| *Eleutherococcus.nodiflorus* | 0 | 0.029153 | 1 | 0.080171 | 0.022675 | 0.29 | 0 | 0.028343 | 1 |
| *Embelia.parviflora* | 0 | 0.043191 | 1 | 0.099441 | 0.02712 | 0.28 | 0 | 0.029129 | 1 |
| *Embelia.ribes* | 0 | 0.127481 | 1 | 0.21204 | 0.147566 | 0.42 | 0.220048 | 0.157042 | 0.2 |
| *Engelhardia.serrata* | 2.336265 | 2.846605 | 0.68 | 4.882181 | 2.86079 | 0.01 | 1.242475 | 2.753525 | 0.94 |
| *Eurya.groffii* | 3.973612 | 5.316851 | 0.72 | 6.270847 | 5.229513 | 0.31 | 5.246027 | 4.944122 | 0.41 |
| *Eurya.impressinervis* | 0.106507 | 0.077379 | 0.6 | 0 | 0.065462 | 1 | 0.109245 | 0.07291 | 0.31 |
| *Eurya.japonica* | 0.102002 | 0.037092 | 0.37 | 0 | 0.029879 | 1 | 0 | 0.035031 | 1 |
| *Eurya.jintungensis* | 0.440863 | 0.380534 | 0.45 | 0.108258 | 0.461392 | 0.96 | 0.797549 | 0.504745 | 0.18 |
| *Fordia.microphylla* | 0 | 0.214473 | 1 | 0.214306 | 0.221396 | 0.45 | 0.449883 | 0.22832 | 0.36 |
| *Fraxinus.chinensis* | 0.315184 | 0.681332 | 0.7 | 0.189053 | 0.726241 | 0.87 | 1.592028 | 0.688691 | 0.08 |
| *Fraxinus.floribunda* | 0.178703 | 0.067691 | 0.13 | 0 | 0.052347 | 1 | 0 | 0.058665 | 1 |
| *Garcinia.cowa* | 0 | 0.026166 | 1 | 0.071956 | 0.023259 | 0.33 | 0 | 0.022532 | 1 |
| *Glochidion.lanceolarium* | 1.674169 | 2.467938 | 0.79 | 3.070324 | 2.616854 | 0.36 | 2.848963 | 2.508663 | 0.34 |
| *Gnetum.montanum* | 0 | 0.769939 | 1 | 2.064047 | 0.811692 | 0.02 | 0.189948 | 0.672364 | 0.94 |
| *Helicia.nilagirica* | 5.664144 | 4.955542 | 0.36 | 8.035354 | 4.44294 | 0.03 | 0.490167 | 4.791184 | 1 |
| *Heliciopsis.henryi* | 0 | 0.067248 | 1 | 0.211352 | 0.071518 | 0.13 | 0 | 0.072585 | 1 |
| *Icacina.oliviformis* | 0.192703 | 0.072994 | 0.19 | 0 | 0.057422 | 1 | 0 | 0.062288 | 1 |
| *Ilex.ficoidea* | 0 | 0.040282 | 1 | 0.107781 | 0.029395 | 0.28 | 0 | 0.038104 | 1 |
| *Ilex.polyneura* | 0 | 0.074934 | 1 | 0 | 0.063486 | 1 | 0.206068 | 0.067649 | 0.09 |
| *Itea.macrophylla* | 0.227742 | 0.198002 | 0.44 | 0.345684 | 0.195866 | 0.37 | 0 | 0.179558 | 1 |
| *Iteadaphne.caudata* | 0.633215 | 0.357282 | 0.13 | 0 | 0.363145 | 1 | 0.433244 | 0.346032 | 0.25 |
| *Lasianthus.chinensis* | 0 | 0.689856 | 1 | 2.107413 | 0.663839 | 0.01 | 0 | 0.753717 | 1 |
| *Lindera.communis* | 0.107442 | 0.042326 | 0.4 | 0 | 0.029302 | 1 | 0 | 0.035814 | 1 |
| *Lithocarpus.fenestratus* | 15.11299 | 15.8086 | 0.48 | 27.53753 | 13.24607 | 0.01 | 0.897851 | 14.4937 | 1 |
| *Lithocarpus.grandifolius* | 0 | 0.517139 | 1 | 1.191071 | 0.460748 | 0.13 | 0.207001 | 0.420184 | 0.53 |
| *Lithocarpus.truncatus* | 5.512193 | 6.763529 | 0.75 | 11.51052 | 6.136429 | 0.01 | 2.026598 | 6.149354 | 1 |
| *Litsea.panamanja* | 1.426577 | 1.119742 | 0.28 | 1.672424 | 1.015014 | 0.15 | 0 | 0.964245 | 1 |
| *Litsea.rubescens* | 2.156957 | 4.746955 | 0.94 | 8.873632 | 4.04098 | 0.01 | 1.665673 | 3.908327 | 0.93 |
| *Lyonia.ovalifolia* | 0.325746 | 5.135113 | 0.95 | 2.049705 | 5.231352 | 0.65 | 13.60321 | 5.612194 | 0.04 |
| *Machilus.nanmu* | 8.275922 | 9.625861 | 0.63 | 16.78579 | 8.820396 | 0.01 | 1.746995 | 8.362451 | 1 |
| *Maesa.japonica* | 0 | 0.195306 | 1 | 0.549818 | 0.181422 | 0.02 | 0 | 0.173091 | 1 |
| *Maesa.ramentacea* | 0.336018 | 0.265136 | 0.43 | 0.44578 | 0.249274 | 0.21 | 0 | 0.267387 | 1 |
| *Melia.azedarach* | 0 | 0.075602 | 1 | 0.202285 | 0.085818 | 0.43 | 0 | 0.040866 | 1 |
| *Melicope.pteleifolia* | 0 | 0.131369 | 1 | 0.376972 | 0.116138 | 0.11 | 0 | 0.129465 | 1 |
| *Michelia.baillonii* | 0.315586 | 0.714835 | 0.83 | 1.670533 | 0.581768 | 0.01 | 0 | 0.689515 | 1 |
| *Michelia.floribunda* | 1.100347 | 0.518458 | 0.01 | 0.32499 | 0.489098 | 0.79 | 0.111113 | 0.528895 | 0.95 |
| *Mucuna* | 0.189364 | 0.138625 | 0.35 | 0.186809 | 0.112027 | 0.35 | 0 | 0.12552 | 1 |
| *Mussaenda.pubescens* | 0.089749 | 0.06918 | 0.41 | 0.088077 | 0.046634 | 0.46 | 0 | 0.062012 | 1 |
| *Myrica.esculenta* | 0.675828 | 1.034826 | 0.74 | 1.011097 | 1.036665 | 0.6 | 1.26055 | 0.875983 | 0.22 |
| *Myrsine.seguinii* | 7.894855 | 7.246402 | 0.41 | 9.183304 | 7.475466 | 0.34 | 4.970762 | 7.327053 | 0.73 |
| *Neocinnamomum.caudatum* | 1.133789 | 0.706118 | 0.2 | 0 | 0.650867 | 1 | 0.924764 | 0.701569 | 0.33 |
| *Olea.europaea.subsp.. Cuspidata* | 0.199817 | 0.080734 | 0.19 | 0 | 0.057523 | 1 | 0 | 0.06156 | 1 |
| *Olea.paniculata* | 0.638092 | 0.702444 | 0.51 | 1.074237 | 0.589965 | 0.23 | 0.210561 | 0.630481 | 0.77 |
| *Olea.rosea* | 3.221281 | 2.064767 | 0.11 | 2.13808 | 2.077015 | 0.4 | 0.675013 | 1.892592 | 0.96 |
| *Phoebe.puwenensis* | 0 | 0.882784 | 1 | 2.412425 | 0.749868 | 0.01 | 0 | 0.779774 | 1 |
| *Photinia.serratifolia* | 0.140579 | 0.493376 | 0.81 | 0 | 0.516547 | 1 | 1.356723 | 0.48738 | 0.03 |
| *Phyllanthus.emblica* | 0.574891 | 1.455074 | 0.7 | 0.226797 | 1.301374 | 0.94 | 3.414258 | 1.459499 | 0.03 |
| *Pinus.kesiya* | 8.738303 | 12.73764 | 0.83 | 0 | 14.16625 | 1 | 32.64696 | 14.48137 | 0.01 |
| *Pittosporum.kerrii* | 0.328443 | 0.219416 | 0.28 | 0 | 0.221149 | 1 | 0.337756 | 0.225635 | 0.08 |
| *Polyspora.chrysandra* | 0.426811 | 1.158717 | 0.75 | 0.214254 | 1.284158 | 0.95 | 3.248454 | 1.446644 | 0.01 |
| *Prunus.serrulata* | 0 | 0.025135 | 1 | 0 | 0.047986 | 1 | 0.113109 | 0.039988 | 0.36 |
| *Psydrax.dicocca* | 0.460439 | 0.748388 | 0.71 | 1.708136 | 0.681274 | 0.04 | 0 | 0.738912 | 1 |
| *Pyrularia.edulis* | 0.104205 | 0.037893 | 0.37 | 0 | 0.03263 | 1 | 0 | 0.033683 | 1 |
| *Pyrus.pashia* | 0.415205 | 0.14679 | 0.11 | 0 | 0.158323 | 1 | 0 | 0.110092 | 1 |
| *Quercus.acutissima* | 0.472095 | 0.175486 | 0.04 | 0 | 0.159273 | 1 | 0 | 0.137337 | 1 |
| *Quercus.aliena* | 0 | 0.495852 | 1 | 0 | 0.708859 | 1 | 1.728499 | 0.523788 | 0.02 |
| *Quercus.griffithii* | 0 | 0.07719 | 1 | 0 | 0.079602 | 1 | 0.238807 | 0.082014 | 0.09 |
| *Quercus.variabilis* | 0 | 0.026162 | 1 | 0 | 0.028778 | 1 | 0.086334 | 0.031394 | 0.37 |
| *Reevesia.rubronervia* | 0 | 0.085257 | 1 | 0.211011 | 0.066074 | 0.08 | 0 | 0.05968 | 1 |
| *Rhododendron.hancockii* | 0 | 1.958599 | 1 | 0 | 1.91272 | 1 | 5.947894 | 2.076574 | 0.07 |
| *Rhus.chinensis* | 0 | 0.112905 | 1 | 0 | 0.137765 | 1 | 0.410186 | 0.159517 | 0.16 |
| *Rubus.lambertianus* | 0 | 0.113808 | 1 | 0.268263 | 0.09484 | 0.36 | 0 | 0.059614 | 1 |
| *Schefflera.heptaphylla* | 0.782493 | 0.496196 | 0.18 | 0.619451 | 0.431205 | 0.31 | 0 | 0.474544 | 1 |
| *Schima.wallichii* | 11.60027 | 16.88096 | 0.92 | 15.15972 | 16.80396 | 0.65 | 23.83751 | 16.91258 | 0.05 |
| *Schoepfia.fragrans* | 0.11207 | 0.12614 | 0.46 | 0.222386 | 0.095752 | 0.18 | 0 | 0.112564 | 1 |
| *Scleropyrum.wallichianum* | 0.54461 | 0.156231 | 0.09 | 0 | 0.195839 | 1 | 0 | 0.192539 | 1 |
| *Staphylea.bumalda* | 0 | 0.026683 | 1 | 0.105664 | 0.042693 | 0.41 | 0 | 0.036289 | 1 |
| *Sterculia.lanceolata* | 0 | 0.032338 | 1 | 0.074452 | 0.019553 | 0.27 | 0 | 0.022561 | 1 |
| *Syzygium.jambos* | 1.188485 | 0.509016 | 0.09 | 0 | 0.453435 | 1 | 0.331936 | 0.557969 | 0.72 |
| *Syzygium.szemaoense* | 0 | 0.237436 | 1 | 0.65295 | 0.184673 | 0.29 | 0 | 0.230841 | 1 |
| *Syzygium.yunnanense* | 0 | 0.061569 | 1 | 0 | 0.081719 | 1 | 0.221648 | 0.07836 | 0.1 |
| *Tarennoidea.wallichii* | 0.910784 | 0.33835 | 0.01 | 0 | 0.262706 | 1 | 0 | 0.309728 | 1 |
| *Ternstroemia.gymnanthera* | 2.538005 | 1.630603 | 0.18 | 1.329575 | 1.191311 | 0.48 | 0.45555 | 1.501215 | 0.89 |
| *Toxicodendron.succedaneum* | 3.867123 | 3.635622 | 0.47 | 0.185202 | 3.663822 | 0.99 | 7.117119 | 3.87 | 0.1 |
| *Turpinia.montana* | 0.211863 | 0.091054 | 0.31 | 0 | 0.102844 | 1 | 0.106338 | 0.124302 | 0.69 |
| *Vaccinium.exaristatum* | 12.26446 | 11.99127 | 0.43 | 13.7604 | 11.64378 | 0.24 | 9.266506 | 11.65631 | 0.72 |
| *Vaccinium.mandarinorum* | 0.23948 | 0.131273 | 0.05 | 0 | 0.132068 | 1 | 0.159136 | 0.135276 | 0.57 |
| *Wendlandia.scabra* | 0 | 0.0685 | 1 | 0.109971 | 0.066525 | 0.52 | 0.113025 | 0.08797 | 0.44 |
| *Wendlandia.tinctoria.subsp.Intermedia* | 2.925338 | 10.94656 | 0.98 | 5.535035 | 11.33299 | 0.89 | 24.90838 | 11.0892 | 0.01 |

***Note：***By Permutation Test, it is verified whether the functional association (ϕ) is significantly higher than the random expectation. p.PKF, p.MCB, and p.MCB are the results obtained from 999 permutation tests. p < 0.05 indicates statistical significance, and there is a non-random functional association among species at this stage of natural recovery.

## Supplementary Figures


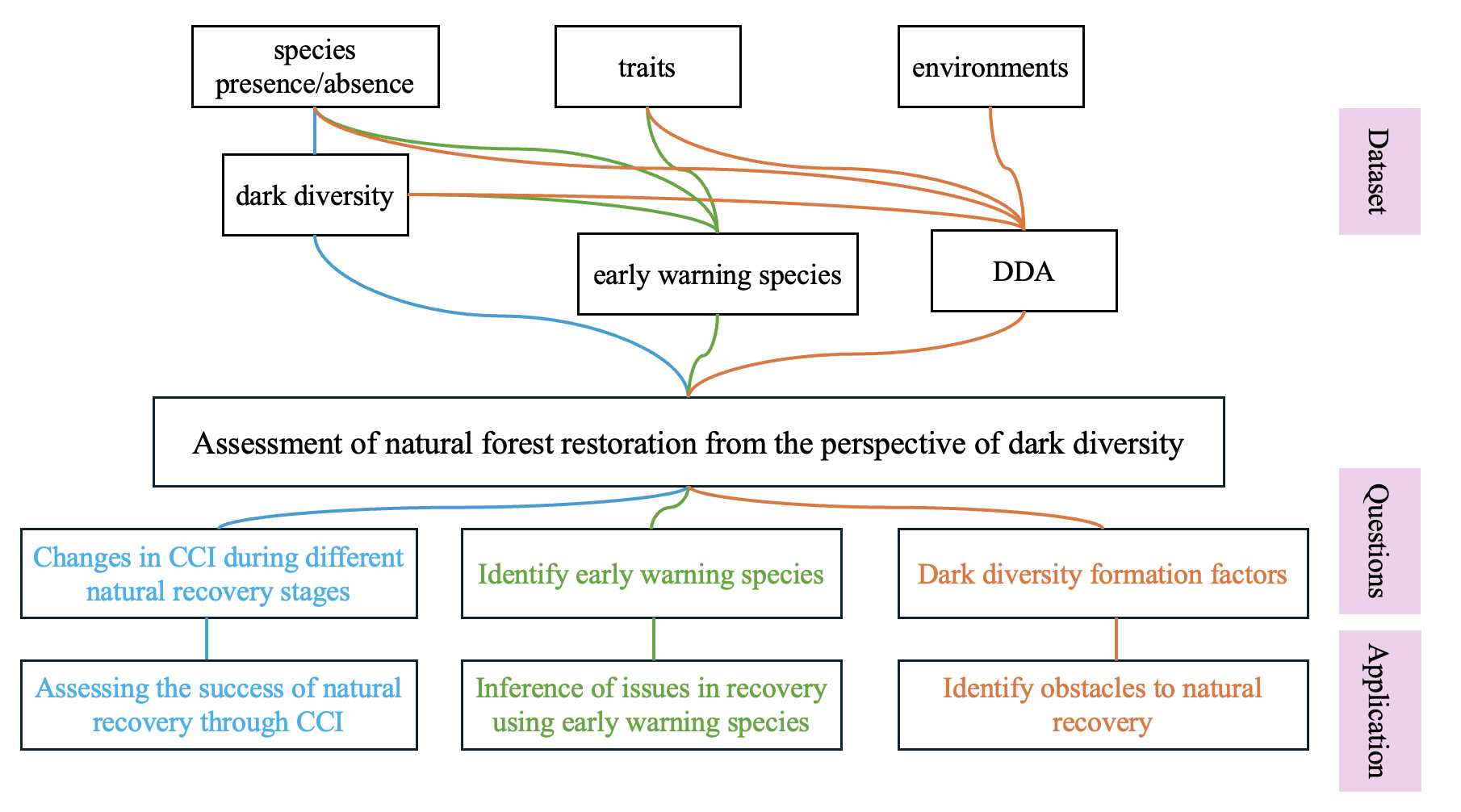


**Supplementary Figure 1.** Datasets and article analysis framework. We used species occurrence data to calculate the observed diversity, dark diversity (species dark diversity and site dark diversity) and community completeness index (CCI). Then analyze the change patterns of CCI during different natural recovery stages to explain how to use CCI to assess whether natural recovery is successful. We use species occurrence data and functional trait data to obtain diagnostic species at different succession stages, combine species dark diversity to determine early warning species. Early warning species can be used to determine the existing problems in the current recovery. Base on species occurrence data , functional trait data, environment data and site dark diversity, we used Bayesian method to quantify the dark diversity affinities (DDA) and decompose it into site dark diversity affinities (dda_site_) and species dark diversity affinities (dda_sp_), thereby identifying the driving factors of dark diversity, that is the factors hindering the natural recovery of forests.


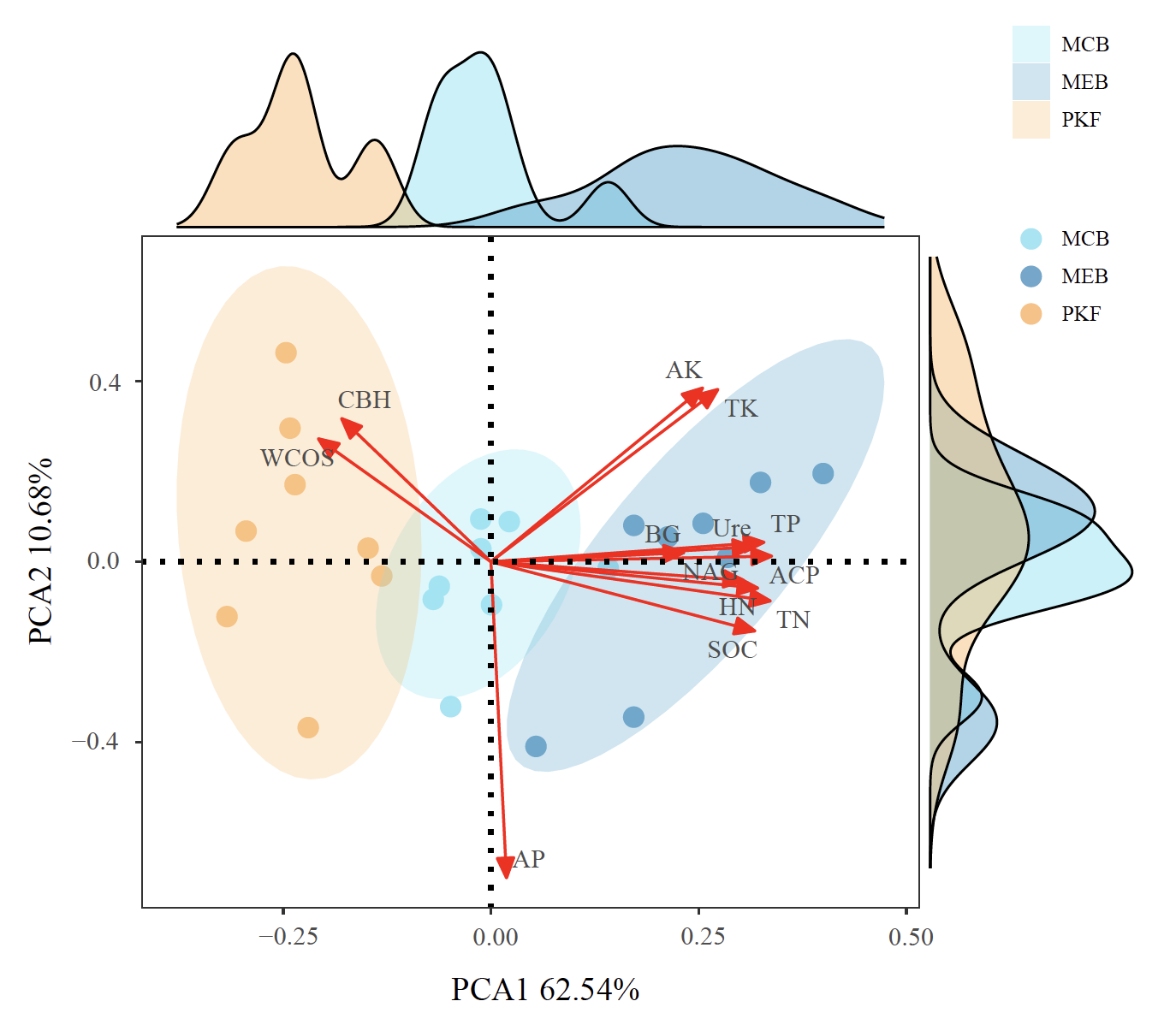


**Supplementary Figure 2.** Results of the soil factor PCA. AP: available phosphorus, AK: available potassium, W: water content, Ure: urease, BG: β-glucosidase, NAG: β-1,4-N-acetylglucosaminidase, CBH: cellulase, ACP: acid phosphatase, SOC: soil organic carbon, TN: total nitrogen, TP: total phosphorus, TK: total potassium, HN: hydrolysable nitrogen.


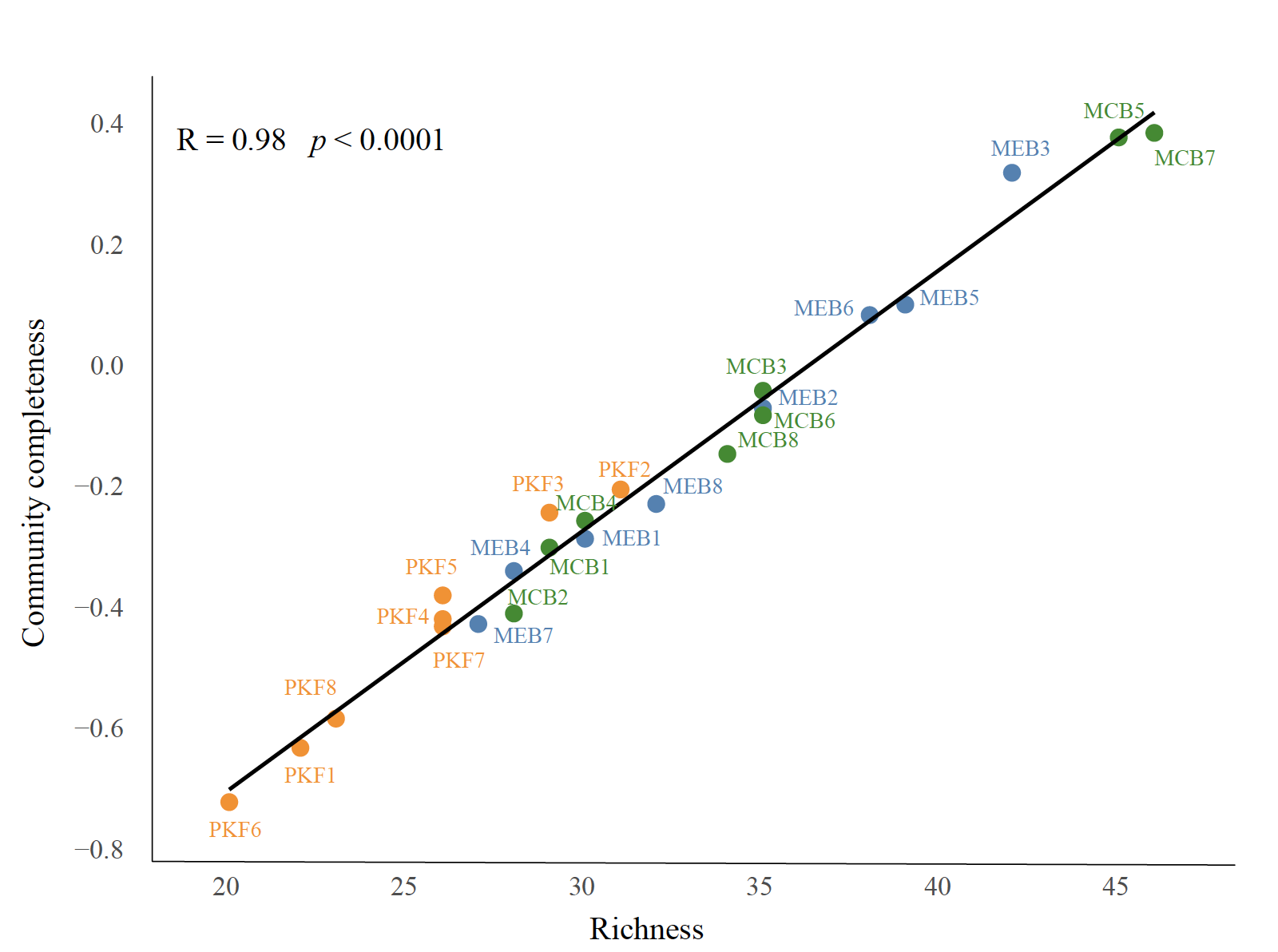


**Supplementary Figure 3.** The relationship between Community completeness index (CCI) and species richness in 24 recovery communities


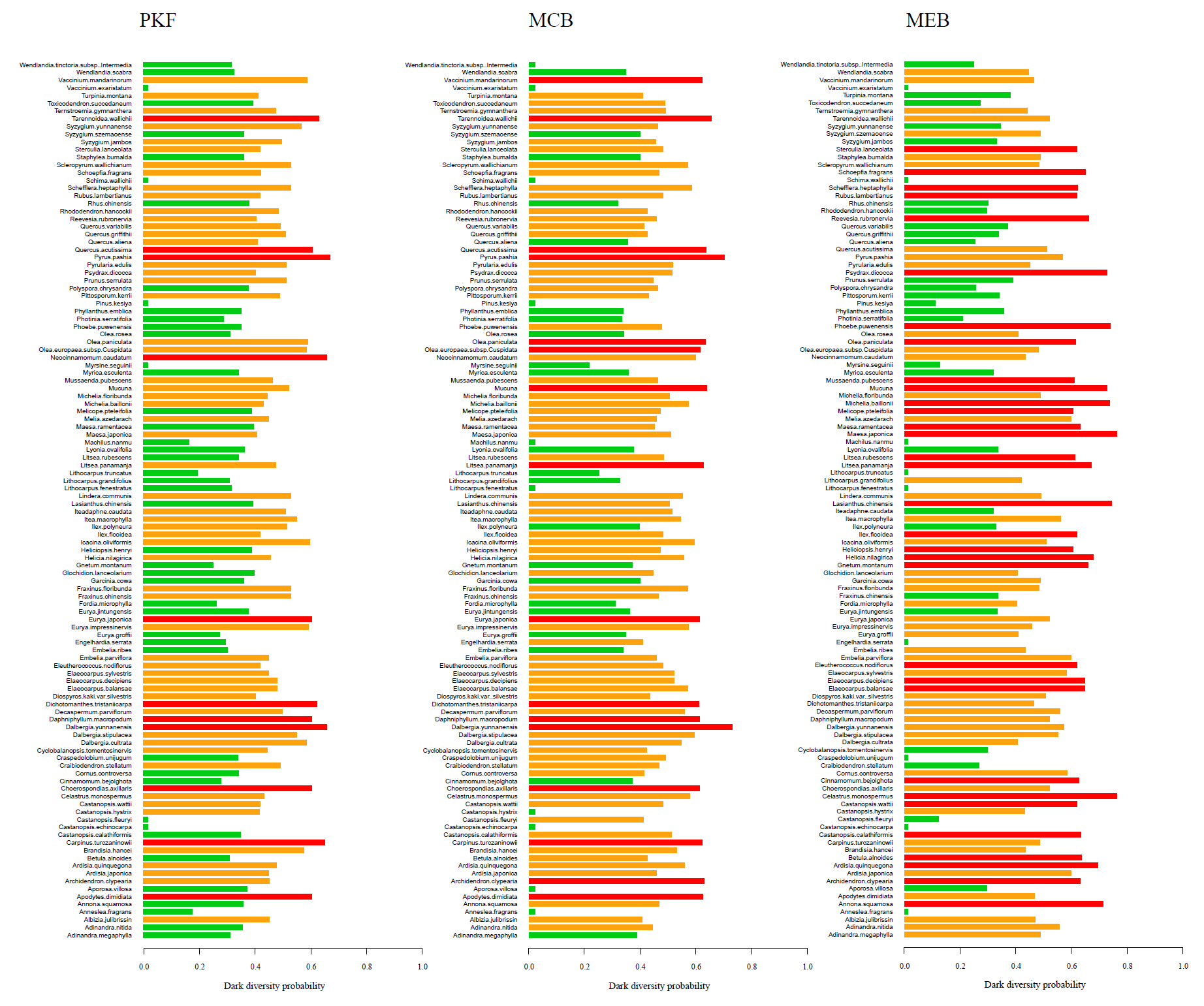


**Supplementary Figure 4.** Species dark diversity at different natural recovery stages.


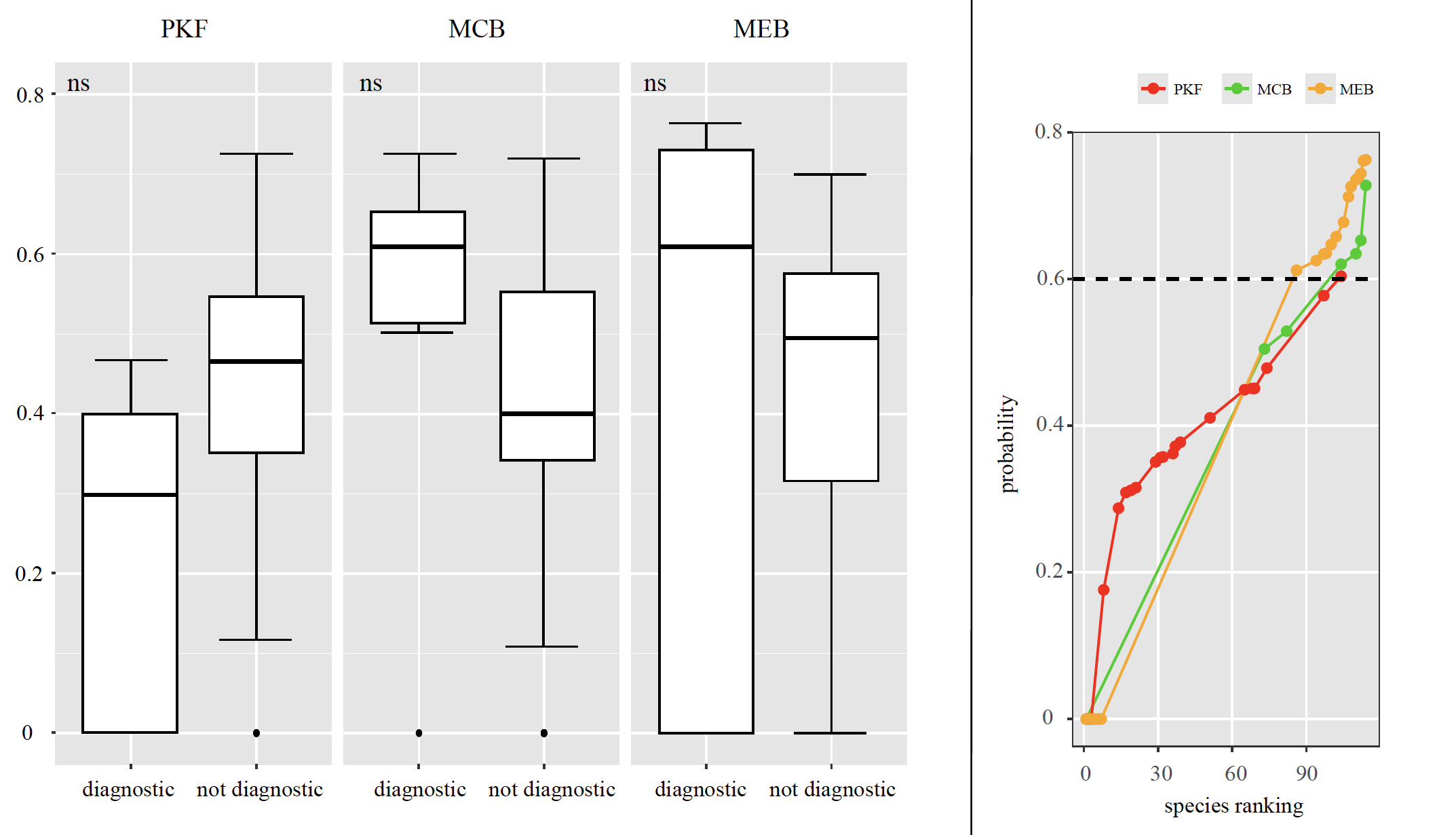


**Supplementary Figure 5.** Analysis of dark diversity differences between diagnostic and non-diagnostic species in different natural recovery stages (left figure). Ranking of species dark diversity in different natural recovery stages (right figure), with the dashed line indicating the dark diversity of 0.6.


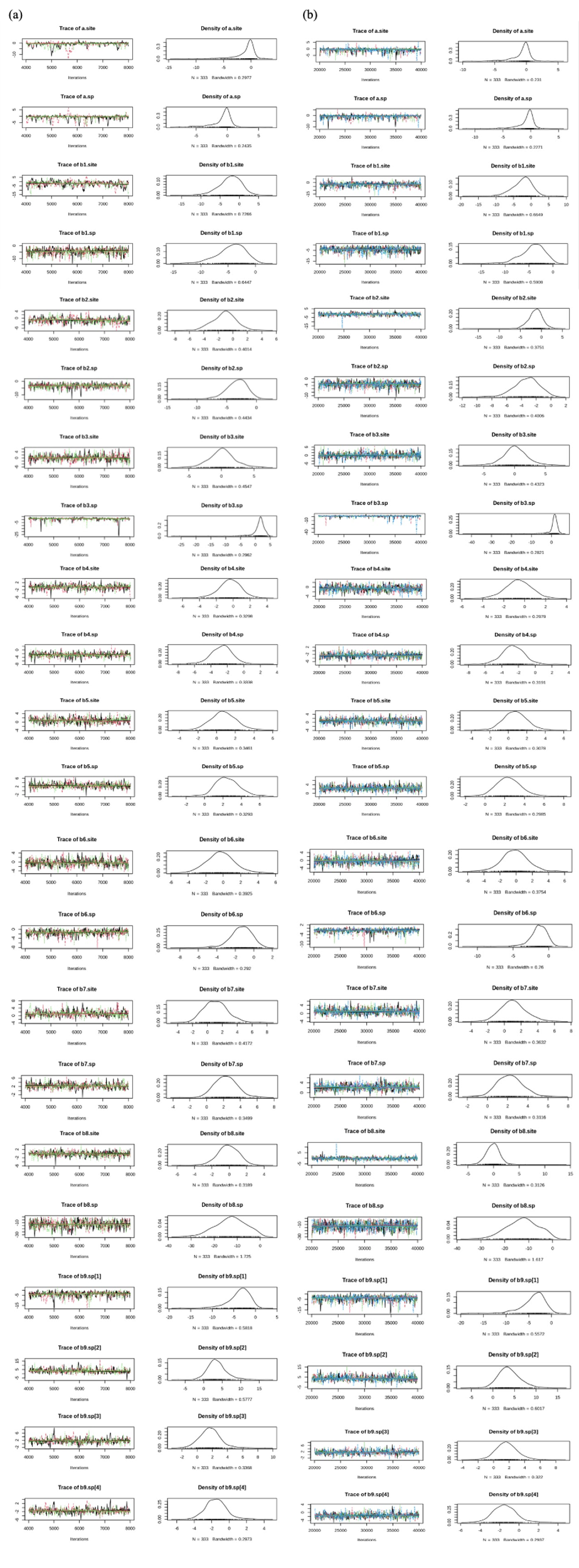


**Supplementary Figure 6.** Diagnostic distributions of the Bayesian model for 4000 (a) and 20000 (b) iterations.
